# Adipocytokine Protein Expression from Visceral Fat Differs Significantly Based on Diet, Sex, and Age in C3H/HeJ Mice Fed Long-Term, High-Fat Diets, ± Ammonium-Hydroxide-Supplemented Dietary Protein

**DOI:** 10.3390/cimb47040218

**Published:** 2025-03-23

**Authors:** Caleb Boren, Benjamin Barr, Noshin Mubtasim, Lauren Gollahon

**Affiliations:** 1 Department of Biological Sciences, Texas Tech University, 2500 Broadway, Lubbock, TX 79409, USA; caleb.boren@ttu.edu (C.B.); benjamin.barr@ttu.edu (B.B.); nmubtasim@gmail.com (N.M.); 2 Obesity Research Institute, Texas Tech University, 2500 Broadway, Lubbock, TX 79409, USA

**Keywords:** adipocytokine, adiponectin, leptin, MCP-1, TIMP-1, Ammonium Hydroxide Enhancement, C3H/HeJ, casein, beef

## Abstract

(1) Background: Overconsumption of processed meats, fats, and carbohydrates drives the obesity epidemic in the USA. Associated with this epidemic are increases in metabolic diseases, such as type 2 diabetes, cardiovascular disease, and cancer. In this study, protein levels of adipocytokines isolated from visceral fat in mice fed high-fat diets with proteins modified through ammonium supplementation were analyzed to determine changes that occur as a result of dietary protein source and its modification based on age or sex. (2) Methods: Male and female C3H/HeJ mice were randomized into six customized diets—Group 1: CCN = Control Chow (CC) + Ammonium Hydroxide Enhancement (AHE); Group 2: CC = Control Chow; Group 3: HFBN = High Fat (HF) AHE Dietary Beef; Group 4: HFB = HF Beef; Group 5: HFCN = HF AHE Dietary Casein; Group 6: HFC = HF Dietary Casein. Mice were censored at six-month intervals, and visceral fat was collected for analysis. This study highlights sex- and age-related changes in cellular adipocytokine protein expression from 12 to 18 months. (3) Results: When compared to dietary casein, dietary-beef-fed mice showed increased expression of adiponectin, leptin, and MCP-1. In dietary casein protein diets, high fat content was correlated with the expression of pro-inflammatory adipocytokines leptin, MCP-1, resistin, VEGF-A, and TIMP-1. Sex-related differences were observed in adiponectin, leptin, and MCP-1 expression levels. AHE of dietary protein decreased the expression of adiponectin, leptin, MCP-1, and TIMP-1. Age-related changes in expression were observed in leptin, MCP-1, and VEGF-A. (4) Conclusions: Our results indicate that the source of dietary protein plays a critical role in determining adipocytokine expression in WAT. Furthermore, this study shows that in addition to dietary protein type (beef or casein), AHE and fat content also impact the relative expression of both pro-inflammatory and anti-inflammatory adipocytokines based on sex over time, with leptin and MCP-1 identified as the most frequently affected.

## 1. Introduction

Based on current studies, it is projected that the population of individuals over the age of 50 in the United States will grow to 221 million by 2050, with over ~143 million reporting at least one chronic disease, such as cardiovascular disease, type 2 diabetes mellitus (T2DM), and cancer [1]. The increase in age-related, non-communicable diseases is tied to the age-associated loss of physiological integrity [2]. Both obesity and low-grade metabolic acidosis have been established as risk factors for the emergence of chronic, non-communicable, age-related diseases, and both are heavily influenced by diet [3,4,5]. Long-term consumption of Western diets rich in saturated fat, calories, and salt with a glycemic index and poor in fiber can cause slight changes in physiological acid base balance [6,7]. This further contributes to the emergence of chronic diseases and the growing burden of deadly clinical outcomes [3,4,7,8,9]. Numerous studies have demonstrated the contribution of obesity in accelerating aging conditions and the incidence of premature death [10,11,12]. Aging is characterized by molecular events, such as telomere shortening, leading to senescence, defective autophagy and mitophagy, an imbalance in reactive oxygen species (ROS), ER stress, a dysfunctional proteasome, apoptosis, and systemic inflammation [8,13,14,15]. Studies also have shown obesity to be a significant risk factor for metabolic acidosis [16].

Metabolic acidosis is a physiological condition of excessive retention of H^+^ ions in the body [6]. Under normal physiological conditions, the primary buffering system “bicarbonate” maintains the normal blood pH [17]. During chronic metabolic acidosis, the kidneys are able to compensate by increasing the production and excretion of ammonia, conserving citrate, and excreting more anions (sulfate, phosphate, and chloride) and minerals (calcium and magnesium) [17,18]. However, detrimental clinical consequences to kidney function occur when they are challenged to maintain homeostasis through continuous activation of these mechanisms. Moreover, maximum excretion of acids per day by the kidneys is ~40–70 mEq, with the excess retained in the body [17]. This problem is due, in part, to a decrease in fiber intake concurrent with an increase in protein and fats, as acidification originates mainly from proteins. However, the likelihood of substantial compliance to a Mediterranean-style diet in the short term is low. To combat the rise of metabolic diseases and attenuate the progression of age-related disorders, it is crucial to identify strategies to control the obesogenic and acidifying potential of the Western diet that are less disruptive to established eating habits in the USA. 

Recent studies have explored a novel approach using ammonium hydroxide to modify dietary proteins (termed Ammonium Hydroxide Enhancement or AHE), with the goal of increasing the overall diet pH to mitigate metabolism-related conditions [9,19,20]. Results from our diet-induced obesity study (DIO), in which C57BL/6 mice were fed a high-fat diet ± AHE, showed improved glucose clearance and serum leptin levels, as well as adiposity [9]. In our long-term study involving DIO in C3H/HeJ mice, results demonstrated improved survivability, improvements in lean and fat mass ratios, attenuation of cancer, and, for beef specifically, improved gut microbiota with AHE [19,20,21].

Studies have established a relationship between obesity and the pathogenicity of accumulated stores of adipose tissue. In metabolism-associated diseases, white adipose tissue (WAT) is the major contributing factor [7]. WAT is an active endocrine organ secreting diverse factors known as adipocytokines. In diets that primarily utilize glucose as the energy substrate, a downregulation of the oxidation of fatty acids occurs, driving the accumulation of fat [7]. Additionally, chronic overconsumption can also shift metabolism to increase fat accumulation. Both of these factors can contribute to the accumulation of WAT that will aberrantly secrete pro-inflammatory adipocytokines that help drive the progression of chronic diseases [15].

Despite the clear connection between obesity and metabolic acidosis, rarely have studies reported findings examining the impact of alternative dietary interventions on the secretion of key adipocytokines from visceral WAT [22]. Furthermore, even less is known about how protein expression of these adipocytokines changes with respect to DIO and aging. The aim of this study was to investigate the effect of AHE dietary protein sources on modulating the expression of key pro-inflammatory and anti-inflammatory adipocytokines in visceral WAT harvested from DIO C3H/HeJ mice over time. Based on our prior reported results [19,20,21,23], we hypothesized that AHE dietary protein would beneficially change the expression profiles of WAT-secreted adipocytokines. To that end, the expression profiles of adipocytokines implicated with chronic disease development [24,25,26] were measured and reported. Overall, this study demonstrates that improvements to protein expression levels from WAT are possible with a dietary intervention incorporating AHE dietary protein sources, without impacting dietary habits.

## 2. Materials and Methods

### 2.1. Mouse Study Design

A total of 400 C3H/HeJ mice, 200 male and 200 female at 4 weeks old, were obtained from Jackson Laboratories (Bar Harbor, ME, USA). Mouse handling followed regulatory compliance at all times under the approved Texas Tech University IACUC protocol number 19021-02 (approval date: 12 February 2019). This strain of mice was selected based on disease susceptibility, lifespan, and less aggressive social behavior [27]. Similarly to our previously reported studies [19,20,21,23], after a week of acclimatization, both the male and female C3H/HeJ mice were randomized into 6 customized diets. Females and males were housed 4 per cage in separate temperature-controlled, humidity-controlled, and 12 h light and 12 h dark cycle-controlled rooms. Animals had access to water and their assigned dietary group in pellet form, ad libitum. The baseline group consisted of 8 male and 8 female mice, which were euthanized after the one-week adjustment period. At this time, the remaining mice were randomly separated by cage into the following diet groups: control casein (CC) (380 kcal/100 g, 11% energy as fat), control casein with AHE (CCN) (380 kcal/100 g, 11% energy as fat), high-fat casein (HFC) (460 kcal/100 g, 46% energy as fat), high-fat casein diet with AHE (HFCN) (460 kcal/100 g, 46% energy as fat), high-fat beef protein (HFB) (470 kcal/100 g, 46% energy as fat), and high-fat beef protein with AHE (HFBN) (470 kcal/100 g, 46% energy as fat). All diets were formulated by Research Diets, Inc. (New Brunswick, NJ, USA). Dietary components are listed in detail in Supplementary Table S6. Mice were maintained on their respective diets for up to 72 weeks. Weekly measurements of individual weights, food consumption per cage, and photographic records of individuals were performed [19,20,21,23]. Monthly measurements to assess changes in fat and lean mass were performed using MRI (EchoMRI, Houston, TX, USA) [19,20,23]. Every 6 months, 8 male and 8 female mice were collected for terminal cross-sectional assessment (censored). At these points, WAT was extracted following methods described previously [23]. At collection time points and at termination of the study, mice were euthanized after a 1 h fast using CO_2_ followed by cervical separation.

### 2.2. WAT Extraction

Following humane euthanasia, murine models were first placed in supine position. Using a scalpel, an incision was made along the midline, extending it caudally to the pubic symphysis. Forceps were used to grasp the skin, and the scalpel was used to carefully separate the skin from the peritoneum. Inside of the peritoneum, perigonadal visceral abdominal white adipose tissue deposits were collected and placed into appropriately labeled 2 mL microcentrifuge tubes. Microcentrifuge tubes containing WAT samples were then flash frozen using liquid nitrogen and stored at −80 °C prior to long-term storage in liquid nitrogen.

### 2.3. Protein Extraction from Adipose Tissue

The protein extraction process was adapted from [28,29]. This process was performed with extreme caution and under low temperatures to prevent protein degradation. Working under low temperatures involved keeping all samples and reagents on ice and working in a cold room set to 4 °C. Reagents used in this process included RIPA Buffer (Thermo Fisher Scientific, Cat#89900, Waltham, MA, USA) and HALT Protease Inhibitor Cocktail (Thermo Fisher Scientific, Cat#87786, Waltham, MA, USA). All reagents used were properly stored at low temperatures, as recommended by the manufacturers, and kept on ice when being used. Moreover, any additional microcentrifuge tubes to be used were stored at −80 °C prior to use and immediately placed on ice after being removed from −80 °C storage. Similarly, when visceral WAT samples were removed from the storage tubes for protein extraction, they were kept on ice. To begin the protein extraction process, ~400 μL of RIPA buffer was added to a 2 mL microcentrifuge tube. For every 100 μL of RIPA buffer, 1 μL of Halt Protease Inhibitor Cocktail was added, as outlined in the manufacturer’s recommendations. After thoroughly vortexing the solution, it was then transferred to a sterile, pre-cooled 2 mL homogenization tube containing beads. An average of ~ 50 mg of sample tissue could be added to the tube before inverting to ensure coating with the RIPA buffer/Halt Protease Inhibitor Cocktail mixture. WAT samples were then homogenized using the Bead Mill Homogenizer (VWR, Cat#432-0367, Radnor, PA, USA), which was preset to 6 m/s for 30 s. Samples were removed and immediately placed on the Barnstead/Thermolyne Labquake Rotisserie Shaker (Ramsey, MN, USA) to allow for continuous agitation for an hour. Following agitation, samples were placed into a MiniSpin Plus (Eppendorf, Hamburg, Germany) microcentrifuge and spun at 13,000 RPM for 15 min. Following centrifugation, the sample separates into a clear supernatant, with the white lipid layer comprising the top. A micropipette was used to pierce the lipid layer and remove the protein extract before transferring it to a new properly labeled 1.5 mL microcentrifuge tube. Protein concentration was then determined prior to final storage at −80 °C to reduce the number of freeze–thaw cycles occurring to preserve protein integrity. 

### 2.4. Protein Quantification

The protein concentration for each sample was then quantified using the Pierce Bicinchoninic Acid (BCA) Protein Assay Kit by Thermo Fisher Scientific (Waltham, MA, USA). The quantification assay was conducted following the manufacturer’s recommendation. Prior to quantification, a portion of each sample was diluted by a factor of ten with RIPA buffer to ensure it fell within the assay’s detectable limits for optimal accuracy. After quantification, the obtained value could be multiplied by the dilution factor to obtain the protein concentration for each sample. 

### 2.5. SDS-PAGE with Coomassie Brilliant Blue Staining

**Figure 1:**
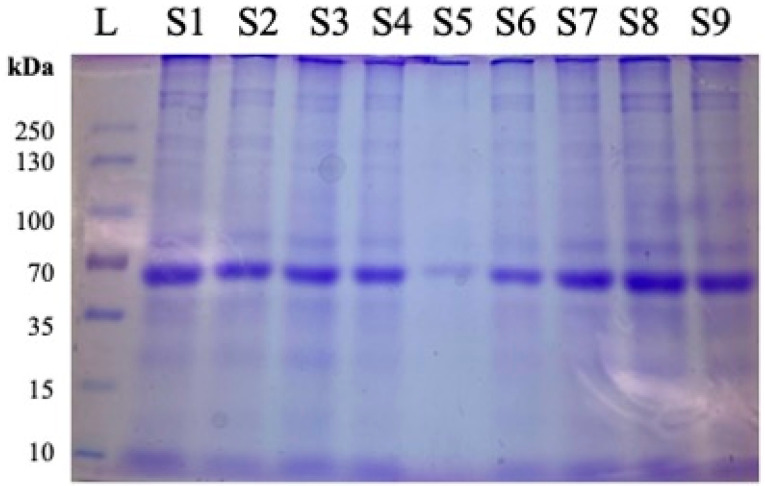
Representative image of protein quality assessment using Coomassie Brilliant Blue after SDS-PAGE and prior to ELISA. Following protein extraction from samples obtained from 12-month females, 20 μg of total protein was added per well for evaluation of protein integrity and loading consistency. L = ladder. Samples are designated as follows: S1 = 1.6.2, S2 = 2.21.2, S3 = 3.38.1, S4 = 4.54.3, S5 = 5.70.3, S6 = 6.85.4, S7 = 1.5.1, S8 = 2.21.4, S9 = 3.37.1, where #.#.# designates Diet Group#, Cage#, Individual Mouse.

Sodium Dodecyl Sulfate Polyacrylamide Gel Electrophoresis (SDS-PAGE) with Coomassie Brilliant Blue staining (Catalog number PI20278, Thermo Fisher Scientific (Waltham, MA, USA)), was performed on every WAT sample to confirm the presence and integrity of the protein. Each sample was equilibrated to a concentration of 20 μg/well for accurate comparison of expression. For the ladder, samples were compared to the PageRuler™ Plus Prestained Protein Ladder, 10 to 250 kDa (Catalog number 26619) by Thermo Fisher Scientific (Waltham, MA, USA). Prior to ELISA analysis of samples, SDS-PAGE with Coomassie Brilliant Blue staining was performed to assess the quality of the extracted proteins before proceeding. Figure 1 shows nine samples being compared against a ladder of known protein weights for an assessment of proteins in WAT lysates. 

### 2.6. ELISA

Enzyme Linked Immunosorbent Assays (ELISAs) were used to determine the presence and concentration of the adipocytokines of interest within samples. Using the data obtained from BCA quantification, the sample concentration was normalized for each ELISA to either 100 μg/well (MCP-1, Resistin, and TIMP-1) or 50 μg/well (Adiponectin, Leptin, and VEGF) depending on the quantity of sample lysates available. The following commercial ELISA kits were used: Mouse Resistin (Invitrogen), Mouse MCP-1 (Invitrogen), Mouse Leptin (Invitrogen), Mouse VEGF-A (Invitrogen), Mouse Adiponectin (Invitrogen), and TIMP-1 (Proteintech). More information regarding the kits utilized can be found in Table 1 below.

### 2.7. Statistical Analysis

LOESS lines were generated using all available measurements of total mass for each week, separated by diet and sex. Using the standard geom_smooth function in ggplot2 (v3.4.2), the SE was generated locally and for each line [30]. Additionally, the mean for each week is presented as points to provide context for the trendlines displayed. No statistical assessment was made. The statistical analyses for ELISA measurements were performed using GraphPad Prism 10 software (GraphPad Software Inc., San Diego, CA, USA). For all samples and analyses, except Group 4 males at 12 months and Group 4 females at 18 months, at least 3 biological replicates were performed with 3 technical replicates. Premature death of mice within those groups prior to censorship prevented the collection of sufficient numbers for ≥3 biological replicates. At 12 months, there were 2 of 3 biological replicates for the Group 4 males. At 18 months, there was 1 of 3 biological replicates for the Group 4 females. To determine the statistical significance, two-way analysis of variance (ANOVA) was utilized. The uncorrected Fisher’s LSD, with a single pooled variance, was used for post hoc analysis due to the unequal sample sizes arising in Group 4, as they were significantly impacted by age-related deaths. A *p* value < 0.05 was considered statistically significant. The sample sizes are n = 3 unless otherwise stated.

## 3. Results

### 3.1. Trends in Total Mass from 12 to 18 Months

**Figure 2:**
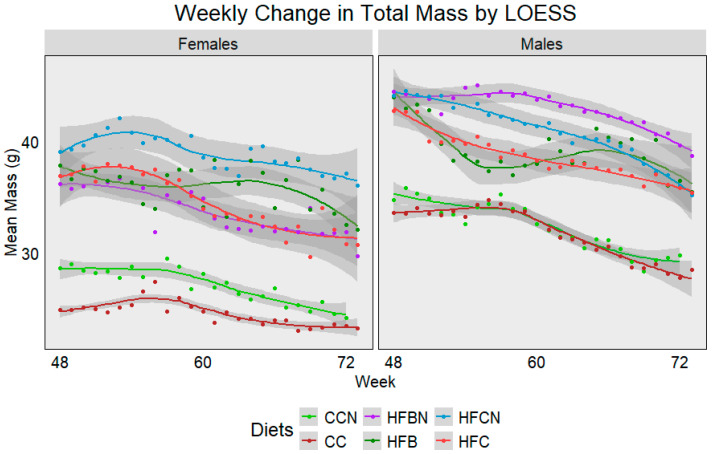
LOESS representations of weekly trends in total mass from 48 weeks (12 months) to 72 weeks (18 months). Colors represent diet groups as follows: light green = control casein AHE (CCN), maroon = control casein (CC), red = high-fat casein (HFC), blue = high-fat casein AHE (HFCN), dark green = high-fat beef (HFB), and purple = high-fat beef AHE (HFBN). Line shadows represent 0.95 CI for the estimation (n = 7–24, based on age).

For reporting purposes, the dietary groups were reported as follows: Control Chow with Ammonium Hydroxide Enhancement (AHE) of Dietary Casein = CCN; Group 2: Control Chow, Non-AHE Dietary Casein = CC; Group 3: High-Fat (HF), Dietary Beef + AHE = HFBN; Group 4: HF, Dietary Beef = HFB; Group 5: HF, Dietary Casein + AHE = HFCN; and Group 6: HF, Dietary Casein (HFC). The average weekly mass from each diet and sex was plotted using Locally Estimated Scatterplot Smoothing (LOESS) (Figure 2). Lines were generated using measurements from all available mice, and dots were added to indicate the true mean total mass for each week. Sample sizes ranged from 24 mice at week 48 to ~7 at week 72 per group. Gray bands indicate SE calculated using R for LOESS. Figure 2 demonstrates a gradual decrease in mass associated with age, regardless of sex, although each diet presents a slightly different sex-dependent profile. In females, HFCN demonstrates the highest total mass, followed by the HFB, HFC, and HFBN, respectively. Both the CC and CCN groups were observed to have an average body mass ~10 g less than those on the high-fat diets, with CCN slightly higher than CC. Interestingly, males exhibit a different total mass profile. For males, HFBN demonstrated the highest total mass, followed by HFCN, HFC, and HFB, respectively. Like the females, the CC and CCN males had average total body mass ~10 g less than those on the high-fat diets, although CC and CCN averages were much closer in the males. It is also important to note that the HFB females and males demonstrated the only increases in total mass occurring with age, but this increase was brief and quickly decreased at ~65 weeks. These data are explored in more depth for 0 to 72 weeks in our previous publications [19,20,21], where body mass composition, microbiota differences, and cancer incidence were also discussed.

### 3.2. Adipocytokine Expression Levels

There is a wide range of both pro-inflammatory and anti-inflammatory adipocytokines that play diverse roles in the development or mitigation of chronic diseases. For this study, to determine adipocytokines that are significantly affected by age, sex, dietary protein source, and/or its modification, our approach was as follows. (1) Conditioned media were first collected from the visceral fat organoid cultures of the C3H/HeJ mice following [23]. (2) To evaluate the secretome of the visceral fat, an adipokine array kit was used to measure protein levels for 38 of the most common adipocytokines. In the Supplementary Materials, Table S1 lists the adipocytokines screened in this array, with those showing significant differences in expression highlighted. Figures S1 and S2 show comparisons between high-fat- and control-diet-fed 12- and 18-month female C3H/HeJ mice, respectively. The data displayed in both Figures S1 and S2 were used to determine which adipocytokines from those analyzed from the adipokine array kit would be studied in more detail. Both adipocytokine protein expression and the response in relation to diet type were key factors in determining which adipocytokines were further analyzed. Thus, adiponectin, leptin, MCP-1, resistin, TIMP-1, and VEGF-A were further analyzed at the cellular protein level through ELISA. While these adipocytokines are well-studied in short-term, in vivo studies [31], there is much less information about long-term changes in expression levels. The results compare adipocytokine levels in more depth individually across all diets and at 12- and 18-month collection points. Complete tables of the measured values of each adipocytokine for both males (Table S2) and females (Table S3) have been provided in the Supplementary Materials. Multifactorial ANOVA results are also included to compare the casein diets (Table S4) and the high-fat diets (Table S5) ± AHE based on time and sex. Supplementary Table S6 lists the components for each different diet.

#### 3.2.1. Adiponectin

**Figure 3:**
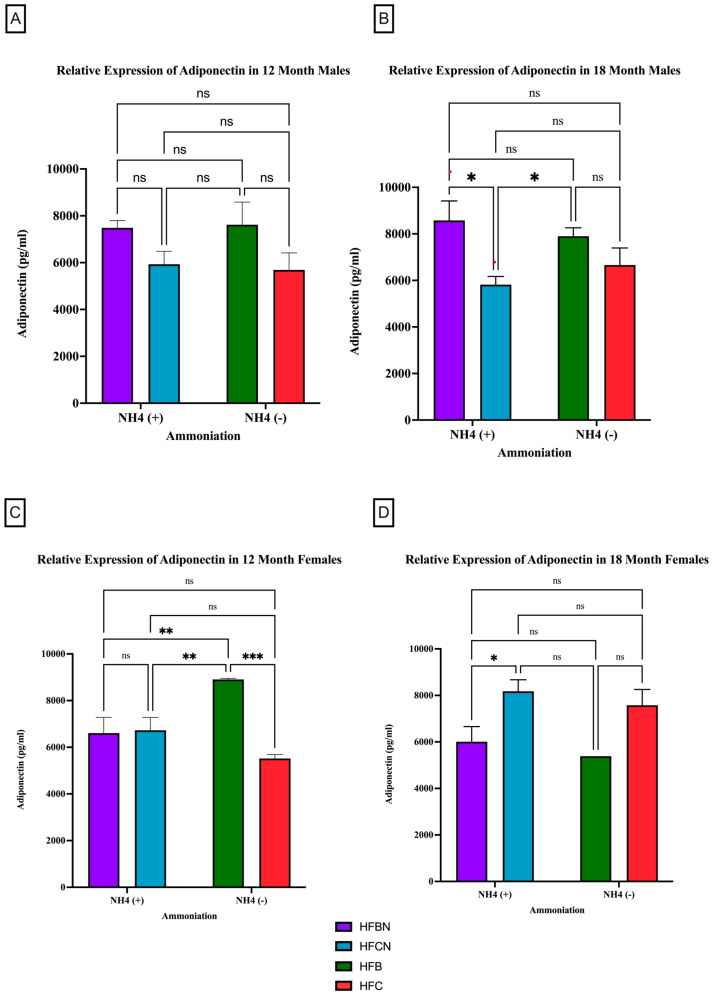
Comparison of the adiponectin protein levels over time among different dietary protein sources ± AHE. Adiponectin protein levels were compared between sex and two different time points (12 months and 18 months) with respect to the effects of the dietary protein source ± AHE. (**A**) Male mice at the 12-month time point. (**B**) Male mice at the 18-month time point. (**C**) Female mice at the 12-month time point. (**D**) Female mice at the 18-month time point. The colors represent diet groups as follows: purple = HFBN, blue = HFCN, green = HFB, and red = HFC. Statistical significance is denoted by ns = no significance; * = *p* < 0.05; ** = *p* < 0.01; *** = *p* < 0.001.

Adiponectin is the most abundantly secreted protein produced by WAT [32]. However, much less is known about protein expression changes. For male mice at the 12-month time point, there was no significant difference observed in the relative expression of adiponectin, irrespective of AHE or protein type (Figure 3A). There was a trend observed for HFB-diet-fed males showing higher relative expression than the HFC-diet-fed group. At 18 months, significant differences between protein types were observed. In the males, HFB ± AHE-diet-fed mice were significantly different from HFCN-diet-fed mice, and when compared to HFC diet results, HFB ±AHE was just outside of the *p* < 0.05 cutoff for significance (Figure 3B).

In contrast to the 12-month males, the 12-month female mice consuming the HFB diets had a significant increase in their relative expression of adiponectin compared to all other diets (Figure 3C). No significant changes in adiponectin expression were found for the female mice between any other diet. The 18-month female results showed the opposite response. Adiponectin in HFBN-diet-fed females was significantly lower than HFCN. While not significant but following the same trend, the HFB-diet-fed females showed markedly less adiponectin than HFC-diet-fed female mice (Figure 3D).

**Figure 4:**
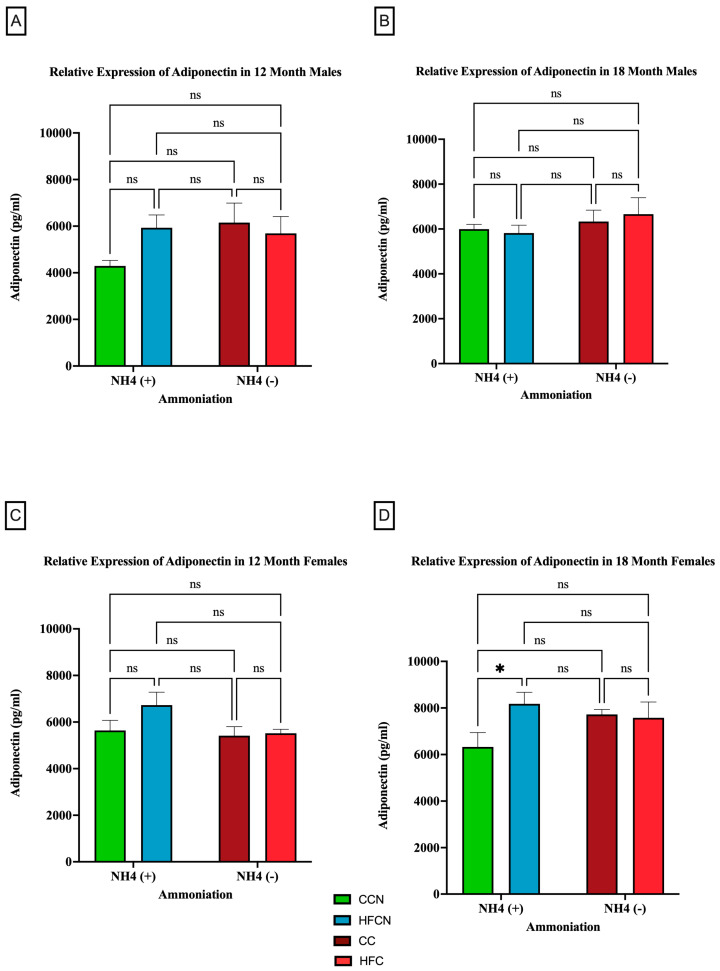
Comparison of the effects of dietary fat content on adiponectin expression. Adiponectin protein levels were compared between sex and two different time points (12 months and 18 months) with respect to the effects of the dietary protein source ± AHE. (**A**) Male mice at the 12-month time point. (**B**) Male mice at the 18-month time point. (**C**) Female mice at the 12-month time point. (**D**) Female mice at the 18-month time point. The colors represent diet groups as follows: green = CCN, blue = HFCN, burgundy = CC, and red = HFC. Statistical significance is denoted by ns = no significance; * = *p* < 0.05.

Figure 4 illustrates the relative expression of adiponectin by AHE and dietary fat content (i.e., HFB/HFBN and HFC/HFCN diets). Despite the lack of significance, a clear difference in sex-based expression of adiponectin can be observed when comparing protein types. In male mice, the 12-month HFCN-diet-fed mice showed higher adiponectin expression than the CCN-diet-fed mice (Figure 4A). Meanwhile, at 18 months (Figure 4B), no differences were evident. The females consuming the HFCN diet show slightly greater expression of adiponectin than those consuming the HFB or HFBN diets (Figure 4C). The only significant difference observed was for 18-month HFCN-diet-fed females, which had significantly higher levels of adiponectin compared to the CCN-diet-fed females (Figure 4D). Interestingly, there was an overall increase in adiponectin expression in females across all diets at 18 months in comparison to 12 months, whereas the males remained relatively constant in expression levels over time.

#### 3.2.2. Leptin

**Figure 5:**
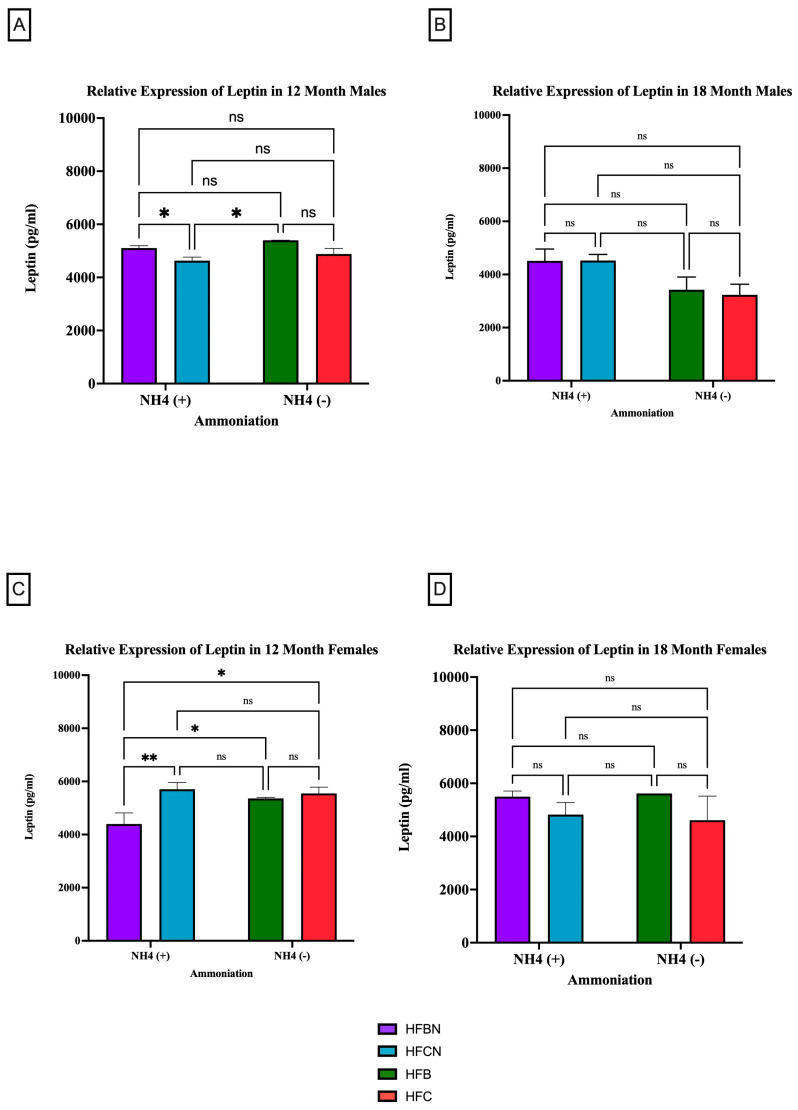
Comparison of the leptin protein levels over time among different dietary protein sources ± AHE. Leptin protein levels were compared between sex and two different time points (12 months and 18 months) with respect to the effects of dietary protein source ±AHE. (**A**) Male mice at the 12-month time point. (**B**) Male mice at the 18-month time point. (**C**) Female mice at the 12-month time point. (**D**) Female mice at the 18-month time point. The colors represent diet groups as follows: purple = HFBN, blue = HFCN, green = HFB, and red = HFC. Statistical significance is denoted by ns = no significance; * = *p* < 0.05; ** = *p* < 0.01.

Leptin is a circulating adipokine that primarily functions to maintain homeostasis between food intake and energy expenditure [33,34]. Figure 5 shows that age has the greatest effect on leptin production as, regardless of sex or diet, there was no significant difference in expression levels. However, at 12 months, it was determined that both sexes experienced a significant change in their leptin expression due to protein type (Figure 5A,C). The males consuming an HFBN diet had an increase in relative expression compared to those on the HFCN diet (5A). At the 18-month time point, there was no significant change in the relative expression of leptin for either sex (Figure 5B,D) in response to enhancement or protein type. Although not significant, in males, leptin is higher in diets containing AHE at 18 months, and, in females, beef seems to slightly increase leptin levels when compared to casein (Figure 5B,D). The females consuming an HFCN diet had an increase in leptin expression compared to those on an HFBN diet. Additionally, the female mice had a significant increase in the expression of leptin for those consuming the HFBN diet instead of the HFB diet.

**Figure 6:**
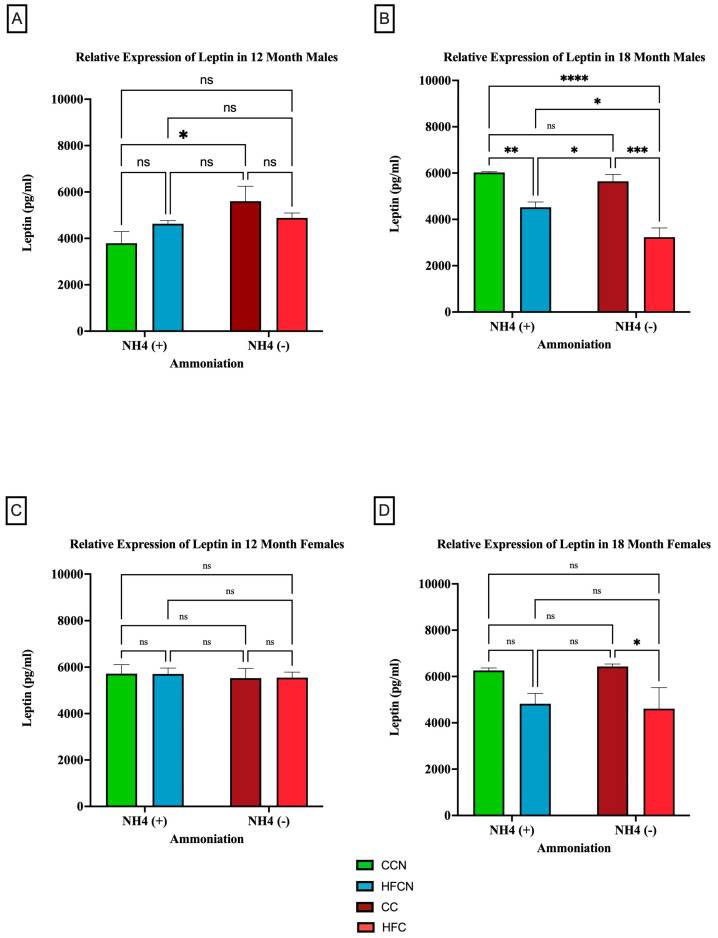
Comparison of the effects of dietary fat content on leptin expression. Leptin protein levels were compared between sex and two different time points (12 months and 18 months) with respect to the effects of dietary protein source ± AHE. (**A**) Male mice at the 12-month time point. (**B**) Male mice at the 18-month time point. (**C**) Female mice at the 12-month time point. (**D**) Female mice at the 18-month time point. The colors represent diet groups as follows: green = CCN, blue = HFCN, burgundy = CC, and red = HFC. Statistical significance is denoted by ns = no significance; * = *p* < 0.05; ** = *p* < 0.01; *** = *p* < 0.001; **** = *p* < 0.0001.

To determine whether dietary fat content influenced leptin expression levels ± AHE, mice fed control diets (with casein as the dietary protein source) and HF casein diets were compared. The results are presented in Figure 6. Males at the 12-month time point who had consumed the CC diet showed higher expression of leptin than those consuming the CCN diet (Figure 6A). At 18 months, the males experienced a significant change in the relative expression of leptin because of the protein type (Figure 6B). Those on the CC diet had higher relative expression levels than those consuming the HFC diet. In addition to protein type, the enhancement of diets with ammonium hydroxide contributed to the change in relative expression of leptin among males. At the 12-month time point, males consuming the CC diet had a higher relative expression of leptin than those on the CCN diet. At the 18-month time point, those males consuming the HFCN diet had a significantly higher relative expression of leptin than those on the HFC diet.

In female mice at both the 12- and 18-month time points, dietary fat content was observed to influence the relative expression of leptin despite not being statistically significant (Figure 6C,D). Those consuming the HFC diets had higher relative expression compared to those on the CC diets.

#### 3.2.3. MCP-1

**Figure 7:**
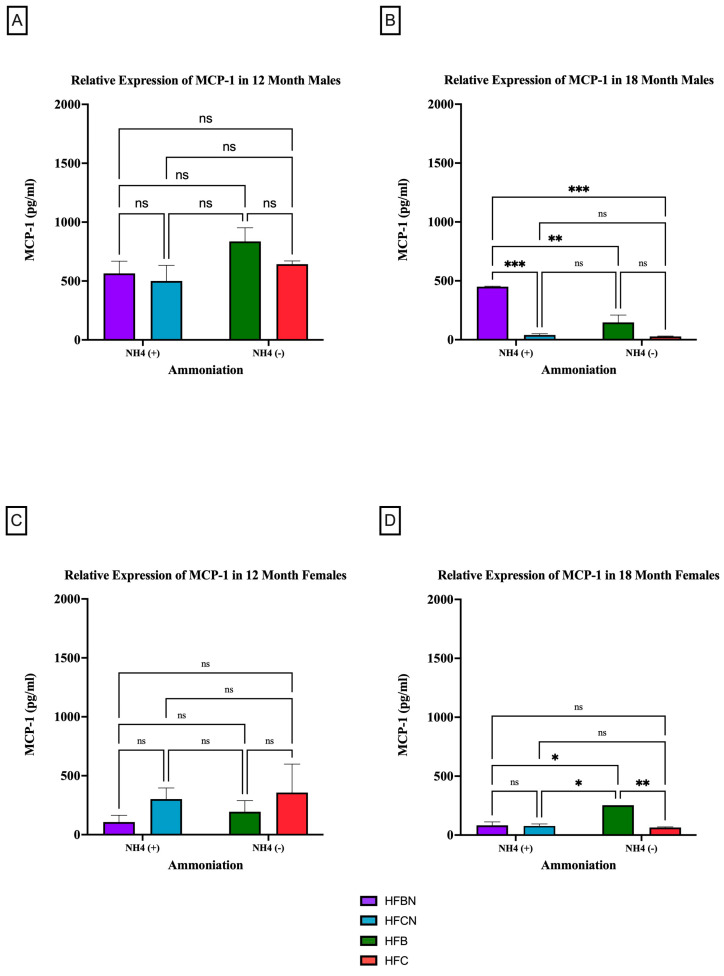
Comparison of the MCP-1 protein levels over time among different dietary protein sources ±AHE. MCP-1 protein levels were compared between sex and two different time points (12 months and 18 months) with respect to the effects of dietary protein source ± AHE. (**A**) Male mice at the 12-month time point. (**B**) Male mice at the 18-month time point. (**C**) Female mice at the 12-month time point. (**D**) Female mice at the 18-month time point. The colors represent diet groups as follows: purple = HFBN, blue = HFCN, green = HFB, and red = HFC. Statistical significance is denoted by ns = no significance; * = *p* < 0.05; ** = *p* < 0.01; *** = *p* < 0.001.

**Figure 8:**
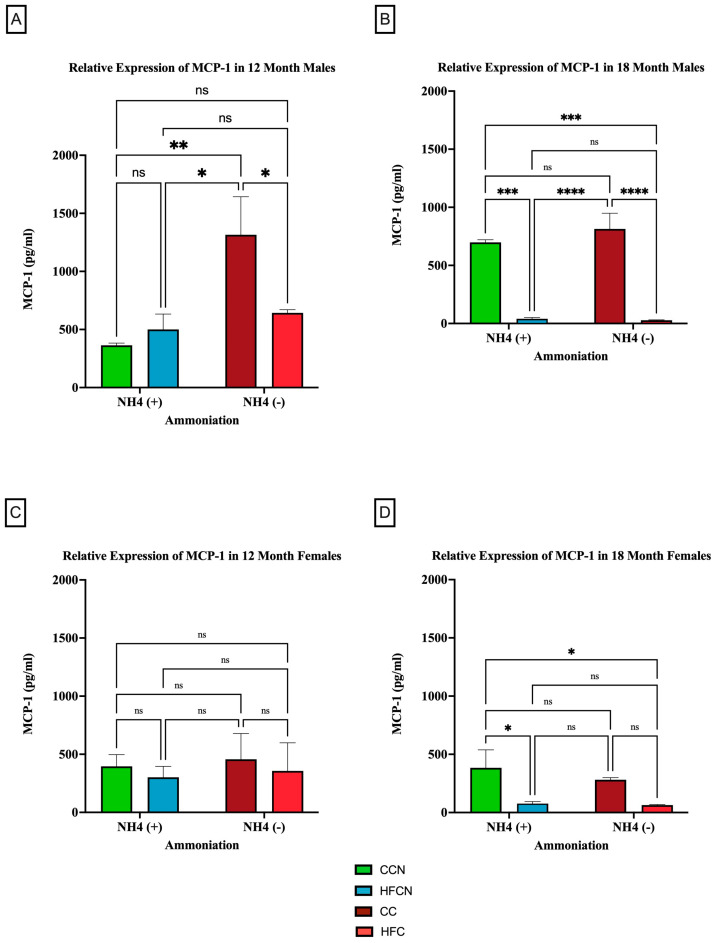
Comparison of the effects of dietary fat content on MCP-1 expression. MCP-1 protein levels were compared between sex and two different time points (12 months and 18 months) with respect to the effects of dietary protein source ± AHE. (**A**) Male mice at the 12-month time point. (**B**) Male mice at the 18-month time point. (**C**) Female mice at the 12-month time point. (**D**) Female mice at the 18-month time point. The colors represent diet groups as follows: green = CCN, blue = HFCN, burgundy = CC, and red = HFC. Statistical significance is denoted by ns = no significance; * = *p* < 0.05; ** = *p* < 0.01; *** = *p* < 0.001; **** = *p* < 0.0001.

MCP-1 is a pro-inflammatory adipocytokine that is known to actively recruit inflammatory cells (i.e., macrophages) to progress the onset of chronic diseases [35,36]. Figure 7 and Figure 8 show results for MCP-1 expression levels across the diets and sex based on dietary protein source ± AHE and aging. The first interesting observation is that, overall, there is markedly more MCP-1 expression in males. At 12 months, there was no significant difference between HF diets regardless of dietary protein source or AHE, although an upward trend was observed in MCP-1 expression levels in the HFB-diet-fed mice (Figure 7A). These results differed greatly from expression levels for the HFD-fed mice at 18 months, where MCP-1 expression levels in casein-based diets were basically nonexistent (Figure 7B). MCP-1 expression in HFBN-diet-fed mice was relatively steady between time points. However, MCP-1 expression in HFB-diet-fed males was significantly lower than at 12 months.

The results for MCP-1 expression in the female mice at 12 months reflected those of the males in that there was no significance between diets regardless of AHE or dietary protein source (Figure 7C). However, the trends reflected by the data were the reverse of the males. MCP-1 was higher in females on a casein-based protein diet. For the female mice at the 18-month time point, there were significant differences identified between HFB and all other diets. HFB-fed females showed the highest relative expression levels for MCP-1. While the measured expression levels appeared to remain very similar between the diets with beef protein, the casein-based protein diet results decreased substantially from 12 months (Figure 7D).

When control fat diets ± AHE (CC, CCN) were compared to HF casein diets ±AHE, the HFCN-diet-fed mice showed statistically significant greater expression levels than all other diets, regardless of fat content or AHE for the males at 12 months. Furthermore, the expression levels in CC- and HFC-diet-fed mice remained statistically similar (Figure 8A). Interestingly, at 18 months, the relative expression analyses showed that the levels of MCP-1 increased in CC-fed mice and concurrently decreased in HFC-diet-fed mice to the point of no significance (Figure 8B). In contrast, the CC and HFC diet groups showed significantly decreased expression at 18 months regardless of AHE (Figure 8B).

When comparing the relative expression levels of MCP-1 in females at both 12 and 18 months, the only reportable differences occurred at 18 months, when CCN-fed females showed significantly higher expression of MCP-1 compared to the HFC-diet-fed groups ±AHE (Figure 8C,D). Of note, the expression levels of MCP-1 for females were lower than those of the males across all diets and time points, in general.

#### 3.2.4. TIMP-1

**Figure 9:**
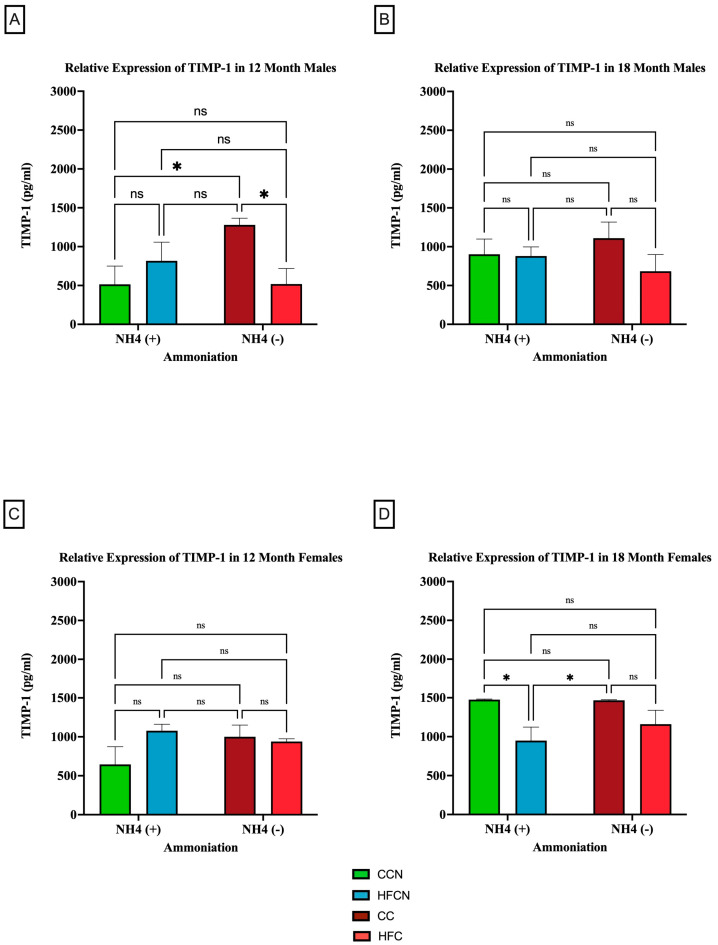
Comparison of the effects of dietary fat content on relative expression levels of TIMP-1. TIMP-1 protein levels were compared between sex and two different time points (12 months and 18 months) with respect to the effects of dietary protein source ± AHE. (**A**) Male mice at the 12-month time point. (**B**) Male mice at the 18-month time point. (**C**) Female mice at the 12-month time point. (**D**) Female mice at the 18-month time point. The colors represent diet groups as follows: green = HFCN, blue = HFCN, burgundy = CC, and red = HFC. Statistical significance is denoted by ns = no significance; * = *p* < 0.05.

TIMP-1 is an endogenous protease that is involved in extracellular matrix remodeling and plays a role in regulating biological processes, such as growth, apoptosis, angiogenesis, and differentiation [37,38,39,40]. Although there were no significant differences in expression between dietary protein sources, AHE status, or time (see Supplemental Figure S3), dietary fat content showed sex-based differences in expression levels and aging (Figure 9). In the males at 12 months, there were significant differences in expression observed between the CC-diet-fed mice compared to CCN- and HFC-diet-fed mice (Figure 9A). No significant differences in TIMP-1 expression were observed at 18 months (Figure 9B).

Interestingly, there was an age-associated, sex-based difference in the effects of dietary fat content in the females. Results from 12 months showed no significance in TIMP-1 expression between diets (Figure 9C). However (and differently from the males), the females at 18 months showed significant expression differences in TIMP-1 between matched AHE pairs. CCN was significantly higher than HFCN-diet-fed mice, and CC was significantly higher in TIMP-1 expression than in HFC-diet-fed mice (Figure 9D). However, the expression levels of matched pairs based on fat content were not significantly different.

#### 3.2.5. Resistin

**Figure 10:**
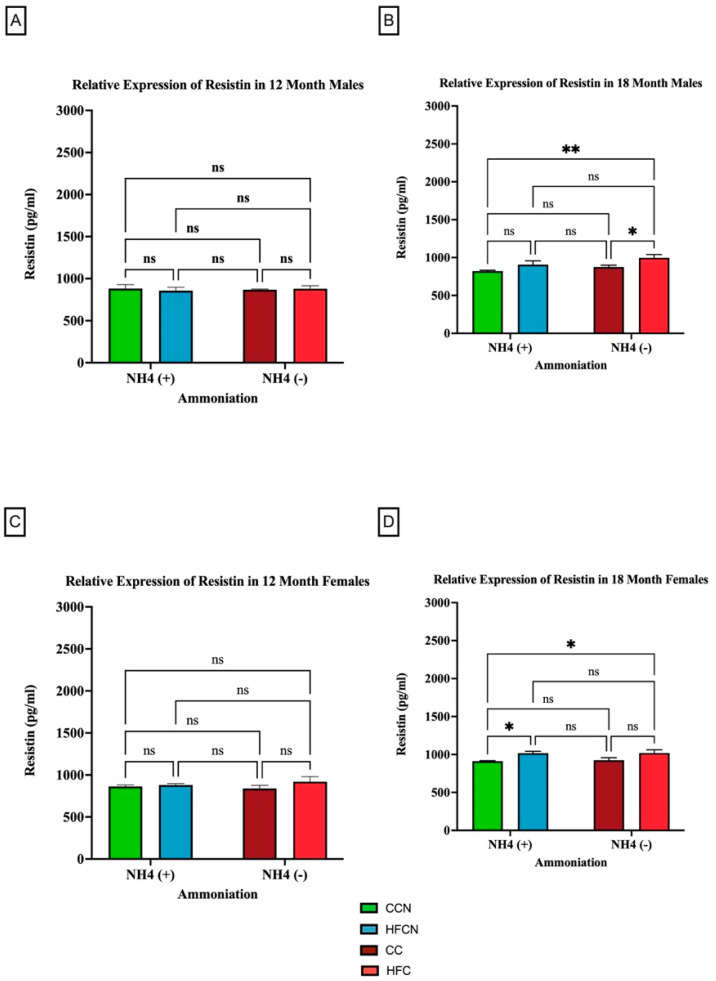
Comparison of the effects of dietary fat content on resistin expression. Resistin protein levels were compared between sex and two different time points (12 months and 18 months) with respect to the effects of dietary protein source ± AHE. (**A**) Male mice at the 12-month time point. (**B**) Male mice at the 18-month time point. (**C**) Female mice at the 12-month time point. (**D**) Female mice at the 18-month time point. The colors represent diet groups as follows: green = CCN, blue = HFCN, burgundy = CC, and red = HFC. Statistical significance is denoted by ns = no significance; * = *p* < 0.05; ** = *p* < 0.01.

Resistin is a pro-inflammatory adipocytokine that is primarily known for its role in insulin resistance [41]. However, studies suggest that it has other roles in association with chronic diseases, such as cardiovascular disease, non-alcoholic fatty liver disease, and kidney disease [42]. Our results show that the most significant factor impacting the relative expression of resistin is fat content (Figure 10). As there was no significance in overall expression of resistin within the HF diets, comparisons for alternate dietary protein sources in HF diets can be found in Supplemental Figure S4. In the 18-month males consuming the non-AHE diets, the mice fed HFC had a higher level of resistin than their CC-diet-fed counterparts (Figure 10B). For females at the same time point, those consuming the HFCN and HFB diet had greater resistin levels than the CCN-diet-fed mice (Figure 10D).

#### 3.2.6. VEGF-A

**Figure 11:**
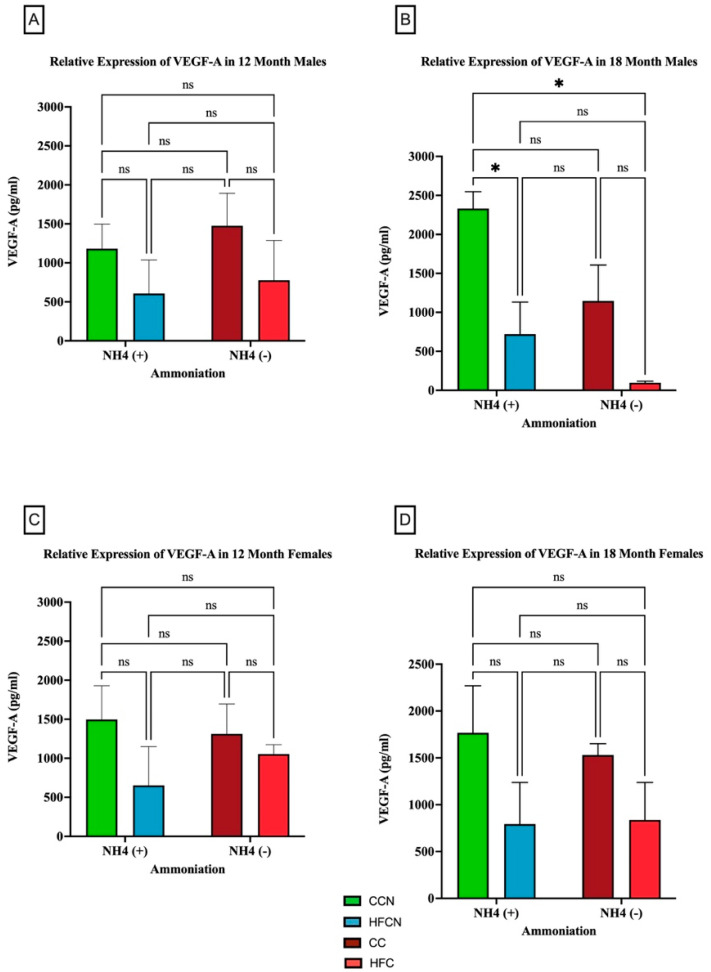
Comparison of the effects of dietary fat content on VEGF-A expression. VEGF-A protein levels were compared between sex and two different time points (12 months and 18 months) with respect to the effects of dietary protein source ±AHE. (**A**) Male mice at the 12-month time point. (**B**) Male mice at the 18-month time point. (**C**) Female mice at the 12-month time point. (**D**) Female mice at the 18-month time point. The colors represent diet groups as follows: green = HFCN, blue = HFCN, burgundy = CC, and red = HFC. Statistical significance is denoted by ns = no significance; * = *p* < 0.05.

The pro-inflammatory adipocytokine VEGF-A has been found to play a critical role in promoting angiogenesis [43]. Interestingly, there were no significant differences in VEGF-A protein expression in the females for any diet. However, our results showed that over time, fat content had a significant impact on the relative expression of VEGF-A (Figure 11). Males consuming the CCN diet at 18 months had the greatest expression of VEGF-A, and it was significant when compared to the decrease in HFCN-diet-fed males. While calculated as statistically not significant, the same pattern was observed for the CC compared to HFC-diet-fed male mice (Figure 11B). In this experiment, dietary beef did not demonstrate a significant difference in the males’ levels of VEGF-A. Data demonstrating no significance from alternate dietary protein sources in HF diets can be found in Supplemental Figure S5.

## 4. Discussion

The association of diet with the development of chronic diseases is the single largest cause of morbidity and mortality in the current world. The presence of high fat content in the diet increases blood acidity [44]. Additionally, according to potential renal acid load values found through different studies and observations, diets high in animal protein release increased amounts of acid precursors into the bloodstream [6,45,46]. Red meat, like beef protein, is slightly acidic, and high fat content can exacerbate the relative acidity. Previous studies have found that high-fat diets significantly increase body and fat mass content [9,31,47] and have an impact on changes in adipokine expression [48]. Recent studies have begun to explore the link between pH modification of dietary protein sources and changes in overall health [9,19,20,21]. The current study further explores the impact of pH modification through AHE of dietary beef and casein in HFD and whether it improves the expression of protective adipocytokines while reducing the expression of harmful adipocytokines as a long-term study (18 months) in C3H/HeJ.

Current preclinical investigations exploring the role of adipocytokines in DIO are generally short-lived. The majority of these studies take place in models that are highly susceptible to DIO and utilize this to rapidly induce obesity in an acute fashion (~16 weeks) [31]. This approach, although more convenient for researchers, likely neglects to provide the nuanced development of obesity as a chronic disease [19]. It has been previously shown that aging has a profound impact on the secretion of adipocytokines by adipose tissue as a result of factors like tissue distribution, the local microenvironment, and sex differences [49]. Additionally, studies have observed age-associated changes in each of the adipocytokines of interest, including adiponectin, leptin, MCP-1, resistin, TIMP-1, and VEGF-A [49,50,51,52,53]. It has been shown that adipocytokine expression is altered based on the amount of fat mass subjects carry, yet few studies have explored how aging, especially loss of fat mass associated with age, impacts adipocytokine expression [48]. Until recently, most studies investigating DIO have not explored it in a sex-specific manner [54,55]. As described by [19], some of the premiere animal models display sex-dependent resistance to DIO, and few studies have explored the factors influencing this or whether some of the same adipokine expression patterns are present.

### 4.1. Adiponectin

Adiponectin is the most abundantly secreted protein produced by white adipose tissue [32]. Despite this, it typically displays an inverse relationship with the total amount of WAT, e.g., adiponectin levels decrease as the total amount of WAT increases [56]. Physiologically, it plays a role in improving metabolism by increasing glucose uptake, inhibiting gluconeogenesis, and enhancing insulin sensitivity, fatty acid oxidation, and anti-inflammation, thereby providing a protective effect against obesity, diabetes, cancer, inflammation, and cardiovascular pathogenesis [32,57,58,59]. Adiponectin has also been shown to play a role in autocrine signaling for tissue differentiation within adipose tissue [60]. Several dietary interventions have been found to have a wide-ranging impact on tissue-specific adiponectin expression. A 16-week in vivo study implementing high-fat and high-sucrose dietary interventions in male mice of the C57BL/6 strain reported obesity and the development of diabetes [61]. The same study observed low expression levels of adiponectin in adipose tissue harvested from the obese and diabetic C57BL/6 mouse strain [61]. Another 16-week dietary intervention study investigating the impact of a high-fat diet on C57BL/6J mice also observed decreased expression of adiponectin in liver and muscle tissue [62]. On the other hand, a two-week dietary intervention study investigating protein diets (15% casein and 5% casein) in Wister rats observed no significant changes in adipokine expression in white adipose tissue [63]. Interestingly, one study found that when subcutaneous adipose tissue growth was uninhibited, it led to increases in intracellular and circulating adiponectin [64]. This finding indicates that obesity-associated deactivation of WAT adiponectin secretion is not only dependent on actual mass but the presence and changes in levels of other signaling molecules, such as leptin [64].

The current study has examined the changes in adiponectin expression in visceral fat tissue harvested from C3H/HeJ mice when fed diets containing HFB or HFC ± Ammonium Hydroxide Enhancement. The AHE of HFB and HFC diets does not have a significant impact on the changes in adiponectin expression in visceral fat harvested from aging C3H/HeJ male mice at 12 months or 18 months. However, the dietary protein source (DPS) does appear to influence the amount of adiponectin measured in males, with beef diets demonstrating elevated adiponectin at 12 and 18 months in comparison to casein diets. Considering adiponectin is a protective adipokine, it is expected that a diet improving overall health may increase adiponectin as part of its benefits. However, the trends in total mass change and the mass composition described in [20] make it more likely that the reduced levels of adiponectin are the result of a reduction in adipose tissue rather than a characteristic of disease [20]. With regards to the female C3H/HeJ at 12 months (~middle age), they showed reduced expression of adiponectin in visceral fat tissue when fed HFBN diets in comparison to females on HFB. The female C3H/HeJ mice fed HFB diet have increased expression of adiponectin at the 12-month time point compared to female mice fed HFC diets. This trend reversed at the 18-month time point, and female mice fed HFC diets have increased relative expression of adiponectin compared to female mice fed HFB diets. Differences in adiponectin levels between HFB and HFC in females suggest that long-term consumption of casein over beef protein may improve adiponectin expression in females. Overall, female mice fed HFC diets were observed to have increased expression of adiponectin compared to female mice fed HFB diets, irrespective of AHE late in life. These observations also correspond to some reported changes in adiponectin discovered through sex-based clinical studies. A longitudinal study led by Chai et al. examining the association of red and processed meat intake with serum adiponectin levels reported lower adiponectin levels in women, with no significant changes in men after nine years of follow-up [65]. A similar study correlating red meat intake with serum adiponectin level led by Ley et.al. in diabetes-free female patients also reported lower concentrations of serum adiponectin with increased red meat intake [66]. Given the protective role of adiponectin expression against metabolic disorder, results from these prior studies and our present study suggest that milk products containing casein protein may provide a protective effect for female C3H/HeJ mice physiology, while beef protein is more protective towards male C3H/HeJ mice physiology. These findings prompt questions concerning the difference in female and male physiologies with respect to lactation, muscle mass, and, possibly, the role of androgens. However, further study is needed to explore these differences. Notably, the differences in expression of adiponectin between the CCN diet and HFCN diet for males at 12 months and females at both time points do not follow the expected trends, where increased mass was also accompanied by increased expression of adiponectin. Coupled with significant differences in aged females, this indicates a potential positive interaction between AHE and HF diets, which may improve the overall metabolic profile by increasing adiponectin expression. Further study is also needed to identify how AHE may interact with an HFD to modify adiponectin signaling from WAT.

### 4.2. Leptin

Leptin is another circulating adipokine produced primarily from white adipose tissue. Its main function is to facilitate the coordination of the physiological balance between food intake and energy expenditure [33,34]. The systemic concentration of leptin increases as the mass of adipose tissue increases [33,67,68,69,70]. The occurrence of high plasma leptin levels increases the risk of progression for various metabolic and inflammatory disease conditions [34,71,72,73,74,75,76,77,78]. Diet has an integral role in the maintenance of plasma leptin concentration. The consumption of high-energy or calorie-dense diets is a leading issue contributing to obesity through the expansion of adipose tissue mass. This can be seen in several research studies that investigated the relationship between calorie-dense diet consumption and leptin expression. From preclinical studies, it was observed that mice fed high-fat diets have an increased concentration of circulating leptin compared to those on a low-fat diet [79,80,81]. However, some data from other clinical and preclinical studies have observed that switching to energy-rich carbohydrate diets has a greater influence on enhancing the level of leptin among subjects than energy-rich, high-fat diets [82,83,84]. These observations suggest that excess consumption of an energy-dense diet has a positive influence on increasing the plasma concentration of leptin. Alas, this direct relationship remains unclear, as other studies have found an inverse relationship of protein diets with the plasma concentration of leptin [85,86,87]. 

In our study, AHE was not observed to significantly alter the expression of leptin in visceral fat harvested from HFD-fed C3H/HeJ mice. Older female mice on HFC ± AHE diets showed increased leptin expression, while males appeared to have increased leptin correlating with HFB ± AHE diets. Furthermore, C3H/HeJ mice demonstrated elevated leptin levels when consuming CC and CCN diets in both sexes. This suggests that with age, the caloric composition of these diets (71% kcal from carbs) provides energy that is more easily utilized than the HFC and HFCN diets. Interestingly, in older males, it appears that AHE increases leptin levels for both DPSs and regardless of dietary fat content. These observations are reflected by the assessments of longevity found in two previously mentioned studies [19,20], especially for the HFBN males, which had markedly improved longevity compared with the HFB. Serum leptin has been linked to fat mass, with increases in serum leptin corresponding to increases in fat mass [88]. However, it is often overlooked when considering weight loss that the function of adipose tissue begins to change. Leptin signaling is more specifically linked to the volume of adipocytes [89]. Thus, it is likely that fat cells are being reduced in volume for the HF diets in association with age, resulting in a decrease in leptin expression overall for C3H/HeJ mice [19]. In contrast, the fat cells in the CC and CCN diets are likely experiencing relatively small changes in volume, while the lean mass has been shown to decrease [19]. In conjunction, this could indicate improved energy availability and bioavailability of the HFBN diets for males. These differences were not as apparent in females. However, this could be attributable to the different needs of each sex with regards to optimal dietary protein sources, hormone signaling, and adipose depot locations [90].

Age-associated changes in the C3H/HeJ mouse model were not observed to significantly impact the expression of leptin in males consuming HFB or HFC diets. However, CC males were found to have a large increase in the expression of leptin associated with aging when compared with HFC males. HFCN-diet-fed females were also found to have a large increase in the expression of leptin. However, with aging, there is a downward trend in the expression of leptin for females fed the HFC diet in comparison to the CC diet. Overall, the study results suggest that CC diet consumption increases the expression of leptin in C3H/HeJ mice more than any of the high-fat protein diets. However, further investigation is needed to determine if this increase is a result of pH enhancement of diet with ammoniation, aging, and/or the genetic makeup of the C3H/HeJ mouse strain.

### 4.3. MCP-1

MCP-1 is a pro-inflammatory adipokine that plays a critical role in the development of many chronic diseases through the active recruitment of other inflammatory signaling molecules and cell types, including, in particular, both monocytes and macrophages [35,36]. In terms of specific immune responses, MCP-1 is involved in signaling pathways, such as p38 MAPK and PI3K. Both of these signaling pathways are important for the downstream modulation of the NF-ĸB pathway, a key inflammatory pathway contributing to various metabolic diseases, such as obesity and metabolic-dysfunction-associated steatotic liver disease. MCP-1 is versatile in its ability to participate in different immune responses depending on the context for its production [35]. Prior in vivo studies have established a connection between the regular consumption of high-fat diets and increased levels of circulating MCP-1, promoting inflammation [91,92]. In our study, at 12 months, increased levels of MCP-1 were observed for mice consuming HFD and AHE compared to those consuming HFD + AHE. The consumption of high-fat diets over time has been linked to the increased generation of reactive oxygen species (ROS), promoting metabolic acidosis [93,94]. This increase in metabolic acidosis can upregulate MCP-1 expression and contribute to the development of chronic disease [35,95]. Dietary interventions could prove to be a valuable resource for reducing the amount of ROS being produced, which in turn would downregulate the expression of MCP-1, reducing its contribution to the development of chronic diseases. Key findings for MCP-1 from this study showed that the dietary protein type contributed to the relative expression of MCP-1. Consumption of a diet with AHE protein modification reduced MCP-1 expression for both male and female mice at 12 months, and male and female mice consuming HF AHE diets had lower levels of MCP-1 expression at 18 months compared to 12 months. This indicates that dietary proteins modified through AHE could be a viable option for reducing the influence of MCP-1-driven inflammation and the progression of chronic diseases.

### 4.4. TIMP-1

TIMP-1 is an adipocyte-derived endogenous protease involved in extracellular matrix remodeling by inhibiting the activity of matrix metalloproteinase [37]. Apart from this, it also plays a role in molecular signaling and can regulate the biological process of growth, apoptosis, angiogenesis, and differentiation [37,38,39,40]. Newer investigations into TIMP-1 show its role as both a regulatory protein and a direct contributor to inflammation [96]. Both IL-1 and IL-6 are inflammatory cytokines that increase the expression of TIMP-1. Additionally, transcriptional influence on TIMP-1 via STAT-3, NF-κB, and AP-1 has been used to link TIMP-1 with an inflammatory response, particularly in the liver [96]. A previous dietary intervention study shed light on how the consumption of a high-fat diet increases the protein levels and secretion of TIMP-1 from visceral fat both in male C57BL/6J and C57BL/6J-Lep^ob^ mice models in comparison to mice consuming a standard chow diet [97]. The study has also reported enhanced serum concentrations of TIMP-1 in response to obesity [97]. In this study, we report related changes in TIMP-1 expression from visceral fat isolated from C3H/HeJ mice through a casein + AHE dietary intervention approach. Interestingly, TIMP-1 expression does not correlate with total mass in this current study’s C3H/HeJ mice, as well as described in studies using other models [97]. In CC males, levels of TIMP-1 were elevated above the other diets, although the CC males had ~10 g less total mass than those on the HF diets and approximately the same total mass as the CCN-diet-fed mice. This is interesting because survivability in this group was much lower than that in mice fed all other diets regardless of DPS [19,20]. Whether or not this finding indicates an issue with the diet (CC) as an option for this mouse strain or a general improvement in health due to AHE is unclear and requires further exploration.

TIMP-1 expression was unique in that expression increased or decreased depending on the presence of AHE and dietary fat. CCN decreased TIMP-1 expression in both males and females at 12 months compared with CC, while in HFCN TIMP-1 it increased compared with HFC. This indicates that AHE-modified dietary protein suppresses TIMP-1 expression in HFD. This observation is crucial as the potential role of pH-centered dietary interventions is explored. It is imperative that each aspect of obesity and other metabolic-associated diseases are examined thoroughly.

### 4.5. Resistin

In this current study, changes in the secretion of the adipocytokine resistin were also observed. Resistin is an adipocytokine-derived endocrine hormone that was originally named for its role in impairing the molecular effect of insulin and inhibiting hepatic gluconeogenesis, resulting in glucose intolerance in rodents [41]. Resistin in excess influences the onset and progression of age-related disorders and chronic diseases through endocrine, paracrine, and autocrine signaling [53,98,99,100]. Mounting evidence from clinical studies has reported high serum resistin levels in patients with metabolically unhealthy obesity, hypertension, diabetes mellitus II, and renal and liver complications [98,100,101,102,103,104,105,106,107,108,109]. In correlating the association of diet with changes in resistin expression, long-term dietary intervention studies with high-protein and high-fat diets have reported significantly increased secretion of resistin from isolated peritoneal adipose tissue with HFD and lower expression with high-protein dietary interventions in comparison to standard diets in a male Wister rat model [110]. Another study compared resistin mRNA expression from isolated WAT with the serum concentration in DIO mice [111]. They reported that although the resistin mRNA expression decreased in WAT, its serum level was markedly elevated in both diet-induced and genetically obese (Lep^ob/ob^), diabetic mice [111]. In the current study, dietary protein type ± AHE did not impact resistin expression levels for either sex over time in the HFD. However, there was a significant difference in resistin expression between AHE diets over time for both sexes, with CCN maintaining the lowest expression levels. Further investigation into the role of resistin will need to consider the possible role of spontaneous TLR-4 mutation, as this has been identified to be a potential confounding factor in C3H/HeJ mice [112,113].

### 4.6. VEGF-A

Adipose tissues are characterized by their lifelong ability to expand [23]. Adipocytes utilize crosstalk to expand when needed to store nutrients in conditions like overconsumption of energy-dense diets and to provide the oxygen necessary for neovascularization [23,26]. WAT secretes an angiogenic biomolecule or protein known as Vascular Endothelial Growth Factor (VEGF) [26]. Both preclinical and clinical data have shown increased expression in the adipose tissue serum concentration of VEGF-A under obese conditions [114,115,116,117]. VEGF-induced neovascularization provides metabolic homeostasis under obese conditions as excess energy from diets accumulates in adipose tissue stores for later use, followed by a reduction in hypoxia, inflammation, and increased protection from the development of insulin resistance [26,118,119,120,121,122]. Because of this, VEGF plays a role in improving adipose tissue function [119,120,121]. Furthermore, there have been reports of decreased expression of VEGF in obese in vivo models [122].

However, obesity-associated increases in the secretion of VEGF have detrimental impacts, as well. It can facilitate the transport of necessary nutrients and metabolites for the growth and proliferation of cancer cells through neovascularization in other tissue stores, which further enhances metastatic potential [123,124,125,126,127]. The current study evaluated changes in VEGF-A expression from visceral fat tissue harvested from male and female C3H/HeJ mice ± AHE dietary intervention in high-fat beef and casein protein diets. The results showed that at 18 months, CCN males had a significantly higher relative expression of VEGF-A than the HFCN males. Due to the highly complex and nuanced nature of VEGF-A signaling, how it interacts with DIO in a chronic setting is yet to be fully understood. Results from this study revealed that a high-fat diet was associated with reduced expression of VEGF-A, which is contrary to studies that show HF-DIO increases the expression of VEGF-A in mouse models [26]. One possibility for this discrepancy is that mouse models in states of acutely induced DIO are studied [19]. In these cases, rather than allowing for chronic increases in fat mass obesity (more reflective of the human condition), obesity is induced over the course of ~12 weeks [19]. Acute induction of DIO likely does not allow fat pads to develop adequate vasculature, resulting in an overall hypoxic condition associated with obesity and disease [120]. However, it has been demonstrated that overexpression of VEGF-A facilitates vascularization of fat pads, resulting in the “browning” of WAT, improving overall metabolic health [120]. Further exploration of VEGF-A in association with chronic DIO is required. Nevertheless, due to trends in total mass before 12 months [19,20], it is possible that VEGF-A expression was increased in association with developing obesity. This sex- and protein-based response is currently under investigation.

### 4.7. Microbiome Changes and High-Fat Diets ± AHE

When discussing differences in adipocytokine expression levels in DIO, the changes in the microbiome should be considered. In our previous publication [21], we examined changes in the microbiome of 16-month-old male and female C3H/HeJ mice. When integrated with results from this study, we found that in aging mice fed a high-fat beef diet, the beneficial mucin-degrader *Akkermansia muciniphila* was dominant, especially in ammonium-supplemented-diet females. Furthermore, other taxa known to be associated with health benefits, including *Romboutsia*, *Oscillospiraceae*, and *Lactococcus cremoris*, were more abundant in ammonium-supplemented-diet microbiomes. Similarly, we identified a range of putatively beneficial microbiome functions associated with ammonium supplementation, such as glycine betaine transport, xenobiotic detoxification, enhanced defense, and others. In contrast, high-fat beef diets were enriched for a set of disease-associated microbiome functions, including functions associated with obesity and metabolic diseases. These outcomes warrant further study of ammonium-supplemented proteins as candidates for improving health by shifting the microbiome. For comprehensive findings of microbiota changes in the HFD-fed mice ± AHE, please see Garrison et al. [21].

### 4.8. Study Limitations

Despite the information this study presents towards better understanding multifactorial interactions between DIO, protein, sex, and aging, there are limitations that should be mentioned. In the statistical analysis, age-related deaths impacting HFB-diet-fed mice resulted in unequal sample sizes. Another limitation is in the identification and quantification of the original 38 adipocytokines. Due to sample size and cost, only females were investigated (primarily because the WAT isolated overlapped with a separate study on breast cancer). The inclusion of males in narrowing down the list of adipocytokines to further investigate may have included others on this list. As previously described, ELISAs were used to determine the relative protein expression. While outside of the scope of this study, RT-qPCR or quantitation of secreted adipocytokine levels would provide a different perspective on changes associated with DIO under these parameters. Additionally, inclusion of mouse plasma serum concentrations of different adipocytokines after AHE dietary intervention at definite time points could also help resolve some of these questions.

## 5. Conclusions

Previous publications from our group showed that AHE has a beneficial impact on the microbiome [21] and attenuated metabolism-associated chronic disease progression [21]. This study focused on investigating the impact of a dietary protein source (beef or casein), its modification using ammonium hydroxide supplementation, and its fat content (control or high-fat) on the relative expression of selected pro- and anti-inflammatory cellular adipocytokines in the context of both sex and age. It was found that the dietary protein source influences the relative expression of these adipocytokines, and the response was different depending on sex and age. It was also found that AHE of dietary protein sources in high-fat diets caused reduced expression of the pro-inflammatory adipocytokines MCP-1 and TIMP-1. Thus, these findings, together with our previous studies, indicate that an AHE-modified dietary protein approach has the potential to help decrease the expression of pro-inflammatory cytokines associated with a HFD without impacting eating patterns. However, this study demonstrates the need to investigate changes in WAT protein expression with sex and aging given the changes we observed based on the dietary protein source and ammonium hydroxide modification of the DPS.

## Figures and Tables

**Table 1:** ELISA and Adipokine Analysis kits used. Provides relevant information regarding the different types of kits that were utilized in this research study.

Assay Kits Applied						
	Kit Type	Species	Protein	Company	Catalog Number	Location
1 *	Adipokine Array	Mouse	38 Common Adipokines	R&D Systems	ARY013	Minneapolis, MN, USA
2	ELISA	Mouse	Adiponectin	Invitrogen	KMP0041	Waltham, MA, USA
3	ELISA	Mouse	Leptin	Invitrogen	KMC2281	Waltham, MA, USA
4	ELISA	Mouse	MCP-1	Invitrogen	BMS6005	Waltham, MA, USA
5	ELISA	Mouse	Resistin	Invitrogen	EMRETN	Waltham, MA, USA
6	ELISA	Mouse	TIMP-1	Proteintech	KE10039	Rosemont, IL, USA
7	ELISA	Mouse	VEGF-A	Invitrogen	BMS619-2	Waltham, MA, USA

* Indicates used in identifying targeted adipokines in a previous publication [23].

## Data Availability

Data are available from the corresponding author upon reasonable request.

## Supplementary Materials

The supporting information can be downloaded at https://www.mdpi.com/article/10.3390/cimb47040218/s1.
