# Supplementary material for: Adipocytokine Protein Expression from Visceral Fat Differs Significantly Based on Diet, Sex, and Age in C3H/HeJ Mice Fed Long-Term, High-Fat Diets, ± Ammonium-Hydroxide-Supplemented Dietary Protein
Source: Curr Issues Mol Biol. 2025 Mar 23;47(4):218. doi: 10.3390/cimb47040218 (PMC12026319; doi:10.3390/cimb47040218)
Supplement: Supplementary file 1 [file cimb-47-00218-s001.zip › cimb-3518267-supplementary.pdf]

Supplementary Materials:

Table S1. – Adipokine array analyses of 38 key adipocytokines associated with obesity and inflammation.

Casein Females	*p-value*		Casein Females	*p-value*	
Age (Months)	12	18	Age (Months)	12	18
Adiponectin	<0.0001	0.2106	Ref spot 1	0.0787	0.0007
DPPIV	>0.9999	>0.9999	Ref spot 2	>0.9999	0.9716
Endocan	>0.9999	>0.9999	Ref spot 3	>0.9999	0.0016
Fetuin A	>0.9999	>0.9999	IL-10	NA	NA
FGF acidic	>0.9999	0.9061	IL-11	NA	NA
FGF-21	0.3744	>0.9999	LIF	NA	NA
HGF	>0.9999	>0.9999	Oncostatin M	NA	NA
ICAM-1	>0.9999	0.9996	Pref-1	NA	NA
IGFBP-3	0.9985	>0.9999	RAGE	NA	NA
IGFBP-6	0.999	0.0002	RANTES	NA	NA
Leptin	0.9958	0.0002	TNF-α	NA	NA
Lipocalin-2	>0.9999	>0.9999	PBS (negative control)	NA	NA
MCP-1	0.9684	<0.0001	AgRP	NA	NA
M-CSF	>0.9999	>0.9999	ANGPT-L1	NA	NA
Pentraxin-3	>0.9999	0.0003	IGF-I	NA	NA
Resistin	<0.0001	0.5775	IGF-II	NA	NA
Serpin E1	0.6444	>0.9999	IGFBP-1	NA	NA
TIMP-1	<0.0001	0.0191	IGFBP-2	NA	NA
VEGF-A	0.0005	0.0386			
RBP4	>0.9999	>0.9999			
IGFBP-5	>0.9999	NA			
IL-6	NA	>0.9999			
Pentraxin-2	NA	>0.9999			
C-reactive protein	NA	>0.9999			

These adipocytokines were previously analyzed [23] (reprinted with permission) in female C3H/HeJ mice, with significant differences highlighted [23]. Of the total adipocytokines examined, 6 were selected for further study.

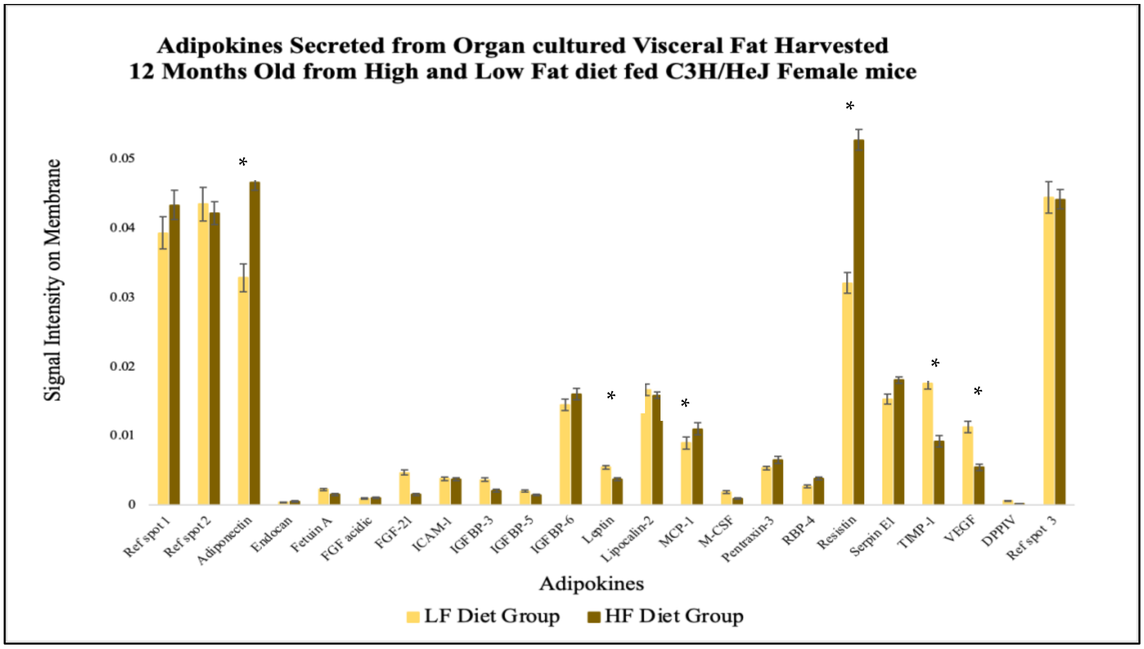

Figure S1. Representative graph for expression differences in 20 adipocytokines at 12-months for control diet-fed female C3H/HeJ mice compared to HFD-fed mice. Cellular protein levels were measured using the Proteome Profiler Mouse Adipokine Array Kit. The colors represent fat content in diet as follows: yellow = LF, Control Diet group -AHE and brown = HF Diet Group -AHE. Three replicates per sample. Statistical significance was determined at p≤0.05.

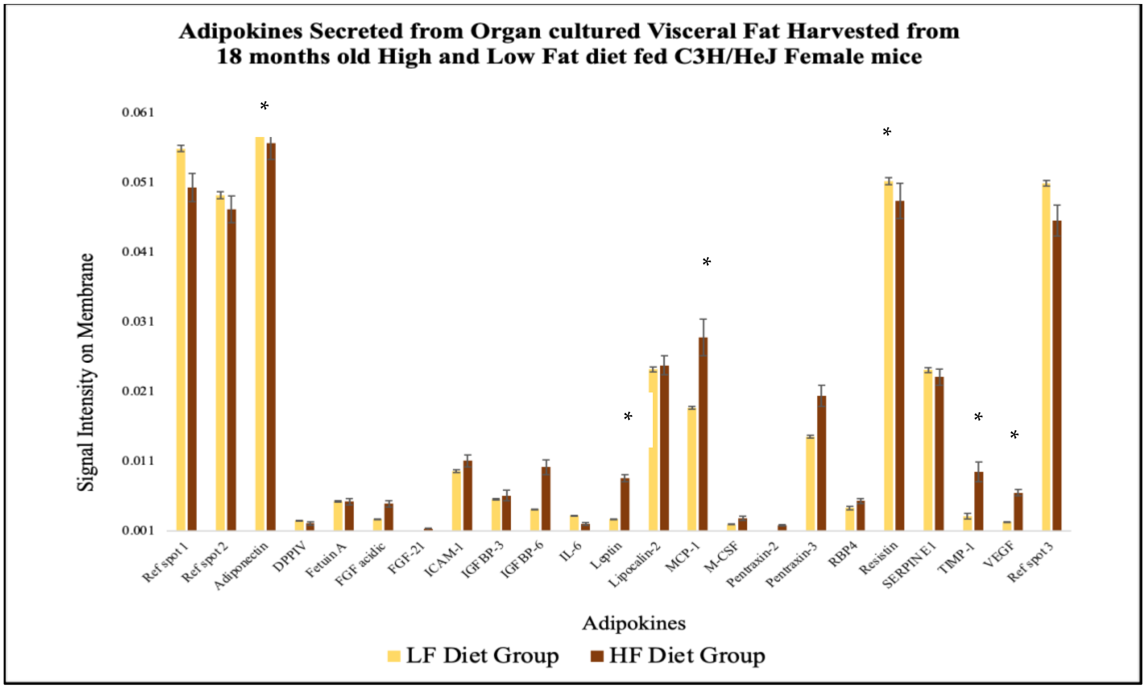

Figure S2. Representative graph for expression differences in 20 adipocytokines at 18-months for control diet-fed female C3H/HeJ mice compared to HFD-fed mice. Cellular protein levels were measured using the Proteome Profiler Mouse Adipokine Array Kit. The colors represent fat content in diet as follows: yellow = LF, Control Diet group

-AHE and brown = HF Diet Group -AHE. Three replicates per sample. Statistical significance was determined at p≤0.05.

Table S2. The expression of pro-inflammatory and anti-inflammatory adipocytokines between the different diet types at 12- and 18-months for male C3H/HeJ mice.

Males	CCN		CC		HFBN		HFB		HFCN		HFC	
Age (Months)	12	18	12	18	12	18	12	18	12	18	12	18
Adiponectin	4294 ± 237.3	5992.9 ± 214.4	6151.8 ± 839.4	6332.9 ± 504.7	7484 ± 310.1	8575.1 ± 839.4	6838.4 ± 960.1	7896.2 ± 364.5	5932.9 ± 549.8	5820.7 ± 350	5688.4 ± 727.5	6660.7 ± 731
Leptin	3787.6 ± 508.4	6028.3 ± 32.5	5601.6 ± 644.6	5644.3 ± 294.3	5106.3 ± 89	4509.6 ± 445.8	5397.6 ± 4	3423.6 ± 477.1	4630.9 ± 132.6	4520.9 ± 231.9	4881.6 ± 212.1	3232.9 ± 397.4
MCP-1	383.1 ± 3.2	697.7 ± 23.1	1315.7 ± 327.2	813.5 ± 134.9	564.5 ± 103.9	299.6 ± 150.8	836.6 ± 115.6	147.3 ± 62.3	500.4 ± 133.1	39.6 ± 10.6	642.8 ± 27.6	27.1 ± 3.6
TIMP-1	515.5 ± 234.8	901.5 ± 196.1	1280.8 ± 85.7	1109.8 ± 207.1	961.2 ± 218.4	814.2 ± 27.7	597 ± 183	650.8 ± 269.7	817.9 ± 239.9	879.2 ± 118.1	519.5 ± 199.8	682.7 ± 215.8
Resistin	874.2 ± 45.5	822.3 ± 10.5	867.9 ± 8.2	875.9 ± 24	787.6 ± 12.6	865.3 ± 62.5	802 ± 30.2	984.1 ± 76	857 ± 40.8	906.8 ± 49.7	879.7 ± 34.5	997 ± 42.2
VEGF-A	1182 ± 315	2332 ± 215	1477 ± 416.1	1147 ± 459.4	843.7 ± 304.3	757 ± 213.8	857 ± 120	413.7 ± 212.2	607 ± 430	720.3 ± 411.4	777 ± 510	97 ± 20

Note: Pro-inflammatory adipocytokines = leptin, MCP-1, TIMP-1, resistin, and VEGF-A. Anti-inflammatory adipokine = adiponectin.

Table S3. The expression of pro-inflammatory and anti-inflammatory adipocytokines between the different diet types at 12- and 18-months for female C3H/HeJ mice

Females	CCN		CC		HFBN		HFB		HFCN		HFC	
Age (Months)	12	18	12	18	12	18	12	18	12	18	12	18
Adiponectin	5638.4 ± 435.6	6326.2 ± 617.1	5414 ± 386.6	7722.9 ± 212.5	6605.1 ± 677.8	6009.6 ± 650.5	8905.1 ± 48	5465.1 ±	6726.2 ± 553.9	8178.4 ± 494.4	5520.7 ± 168.8	7576.2 ± 680.1
Leptin	5718.3 ± 385.8	6258.9 ± 108.5	5524.9 ± 422.4	6430.3 ± 107.8	4396.3 ± 417.4	5496.9 ± 215.4	5358.9 ± 39.8	5619.6 ±	5706.3 ± 255	4824.3 ± 448.3	5548.3 ± 235.3	4609.6 ± 911
MCP-1	396.1 ± 101.3	384.9 ± 154.1	457.1 ± 222.1	282 ± 18.5	108.6 ± 56.7	82.6 ± 29.8	194.3 ± 94.2	253.9 ±	301.8 ± 94.4	76.7 ± 17.7	357.3 ± 242	37.3 ± 27.4
TIMP-1	644.7 ± 226.8	1476.2 ± 7.5	999.4 ± 150.1	1467.3 ± 7.4	799.4 ± 219.3	1297.9 ± 27.1	863.2 ± 20.6	1421.4 ±	1077.4 ± 83.5	949.4 ± 173.3	938.5 ± 35.1	1160.8 ± 178.5
Resistin	862.4 ± 19.1	911.8 ± 5.8	838.8 ± 38.1	924.7 ± 32.4	847 ± 40.1	965.5 ± 50.9	861.1 ± 25.4	979.5 ±	880.8 ± 16	1018.3 ± 23.5	920.5 ± 59.9	1018.9 ± 43.2
VEGF-A	1497 ± 431.3	1767 ± 502.1	1313.7 ± 381.1	1530.3 ± 121.4	1117 ± 890	367 ± 70	552 ± 435	1797 ±	653.7 ± 497	793.7 ± 444.1	1053.7 ± 121.2	837 ± 400.7

Note: Pro-inflammatory adipocytokines = leptin, MCP-1, TIMP-1, resistin, and VEGF-A. Anti-inflammatory adipokine = adiponectin.

Table S4 – Multifactorial ANOVA results of interactions between AHE and Dietary Fat Content of a Casein Diet.

Casein Diets			Main Effects			Interactions
Variables						
Adipokine	Age (Months)	Sex	Statistic	Enhancement (E)	Fat Content	E x F
Adiponectin	12	Males	*p*	0.2371	0.3788	0.1344
			F (1, 8)	1.633	0.8678	2.773
	12	Females	*p*	0.1198	0.1838	0.2663
			F (1, 8)	3.033	2.117	1.428
	18	Males	*p*	0.2623	0.8776	0.6232
			F (1, 8)	1.454	0.02527	0.2611
	18	Females	*p*	0.4768	0.1477	0.0972
			F (1, 8)	0.5571	2.568	3.527
Leptin	12	Males	*p*	0.0428	0.8893	0.106
			F (1, 8)	5.787	0.02065	3.318
	12	Females	*p*	0.6137	0.9869	0.9592
			F (1, 8)	0.2758	0.0002869	0.002789
	18	Males	*p*	0.0157	<0.0001	0.1371
			F (1, 8)	9.338	51.3	2.73
	18	Females	*p*	0.9674	0.0132	0.7167
			F (1, 8)	0.001781	10.05	0.1413
MCP-1	12	Males	*p*	0.015	0.1688	0.0519
			F (1, 8)	9.512	2.289	5.206
	12	Females	*p*	0.7525	0.6005	0.9877
			F (1, 8)	0.1065	0.2972	0.0002527
	18	Males	*p*	0.4733	<0.0001	0.3771
			F (1, 8)	0.5662	0.5662	0.8745
	18	Females	*p*	0.5367	0.0209	0.6242
			F (1, 7)	0.4219	8.801	0.2625
TIMP-1	12	Males	*p*	0.2766	0.2844	0.0288
			F (1, 8)	1.363	1.316	7.073
	12	Females	*p*	0.4731	0.2308	0.1233
			F (1, 8)	0.5667	1.682	2.966
	18	Males	*p*	0.9758	0.267	0.3138
			F (1, 8)	0.0009815	1.424	1.155
	18	Females	*p*	0.4397	0.0101	0.4022

Adipokine	Age (Months)	Sex	Statistic	Enhancement (E)	Protein (P)	E x P
			F (1, 8)	0.6611	11.2	0.7825
Resistin	12	Males	*p*	0.9039	0.8686	0.6346
			F (1, 8)	0.01554	0.02918	0.244
	12	Females	*p*	0.8333	0.2212	0.4214
			F (1, 8)	0.04727	1.76	0.7182
	18	Males	*p*	0.0749	0.0191	0.6169
			F (1, 8)	4.189	8.562	0.2707
	18	Females	*p*	0.8249	0.0095	0.8402
			F (1, 8)	0.05227	11.5	0.04341
VEGF-A	12	Males	*p*	0.621	0.2084	0.893
			F (1, 5)	0.2772	2.084	0.02003
	12	Females	*p*	0.7856	0.1898	0.4704
			F (1, 8)	0.07917	2.053	0.5739
	18	Males	*p*	0.0676	0.0169	0.5151
			F (1, 6)	4.957	10.74	0.4782
	18	Females	*p*	0.8129	0.068	0.7323
			F (1, 8)	0.05984	4.447	0.1255

Table S5. Multifactorial ANOVA results for interactions between AHE and Dietary Protein Source in a HF Diet.

HF Diets			Main Effects			Interactions
Variables						
Adipokine	Age (Months)	Sex	Statistic	Enhancement (E)	Protein (P)	E x P
Adiponectin	12	Males	*p*	0.9334	0.0282	0.7726
			F (1, 7)	0.007497	7.601	0.09024
	12	Females	*p*	0.2553	0.0064	0.0044
			F (1, 8)	13.36	1.502	15.42
	18	Males	*p*	0.8984	0.0115	0.2492
			F (1, 8)	0.01737	10.65	1.544
	18	Females	*p*	0.4465	0.0274	0.9898
			F (1, 6)	0.6633	8.401	0.000177
Leptin	12	Males	*p*	0.1149	0.0133	0.8964
			F (1, 7)	3.241	10.84	0.01824
	12	Females	*p*	0.1775	0.0249	0.0735
			F (1, 8)	2.186	7.59	4.24
	18	Males	*p*	0.0178	0.828	0.8067

			F (1, 8)	8.833	0.05041	0.06395
	18	Females	*p*	0.9521	0.2953	0.8259
			F (1, 6)	0.003928	1.314	0.05281
**MCP-1**	12	Males	*p*	0.087	0.255	0.5537
			F (1, 7)	3.957	1.537	0.3868
	12	Females	*p*	0.6268	0.2419	0.9125
			F (1, 8)	0.2556	1.597	0.01286
	18	Males	*p*	0.0032	0.0002	0.0049
			F (1, 7)	19.3	54.59	16.35
	18	Females	*p*	0.5367	0.0209	0.6242
			F (1, 7)	0.4219	8.801	0.2625
**TIMP-1**	12	Males	*p*	0.183	0.6373	0.8874
			F (1, 7)	2.184	0.2428	0.02158
	12	Females	*p*	0.7604	0.1762	0.4193
			F (1, 8)	0.09955	2.201	0.7248
	18	Males	*p*	0.3542	0.7978	0.9305
			F (1, 8)	0.9672	0.07015	0.008109
	18	Females	*p*	0.3806	0.136	0.8123
			F (1, 6)	0.8954	2.962	0.06159
**Resistin**	12	Males	*p*	0.5915	0.0584	0.9058
			F (1, 7)	0.3161	5.104	0.01505
	12	Females	*p*	0.5095	0.267	0.7481
			F (1, 8)	0.4766	1.424	0.1105
	18	Males	*p*	0.1145	0.6571	0.8146
			F (1, 8)	3.137	0.2125	0.05874
	18	Females	*p*	0.8883	0.3919	0.8974
			F (1, 6)	0.02147	0.8509	0.0181
**VEGF-A**	12	Males	*p*	0.813	0.6846	0.8397
			F (1, 5)	0.06218	0.1855	0.04541
	12	Females	*p*	0.8732	0.9704	0.3676
			F (1, 6)	0.02774	0.001497	0.949
	18	Males	*p*	0.0032	0.0002	0.0049
			F (1, 7)	19.3	54.59	16.35
	18	Females	*p*	0.188	0.6049	0.2109
			F (1, 5)	2.323	0.3044	2.058

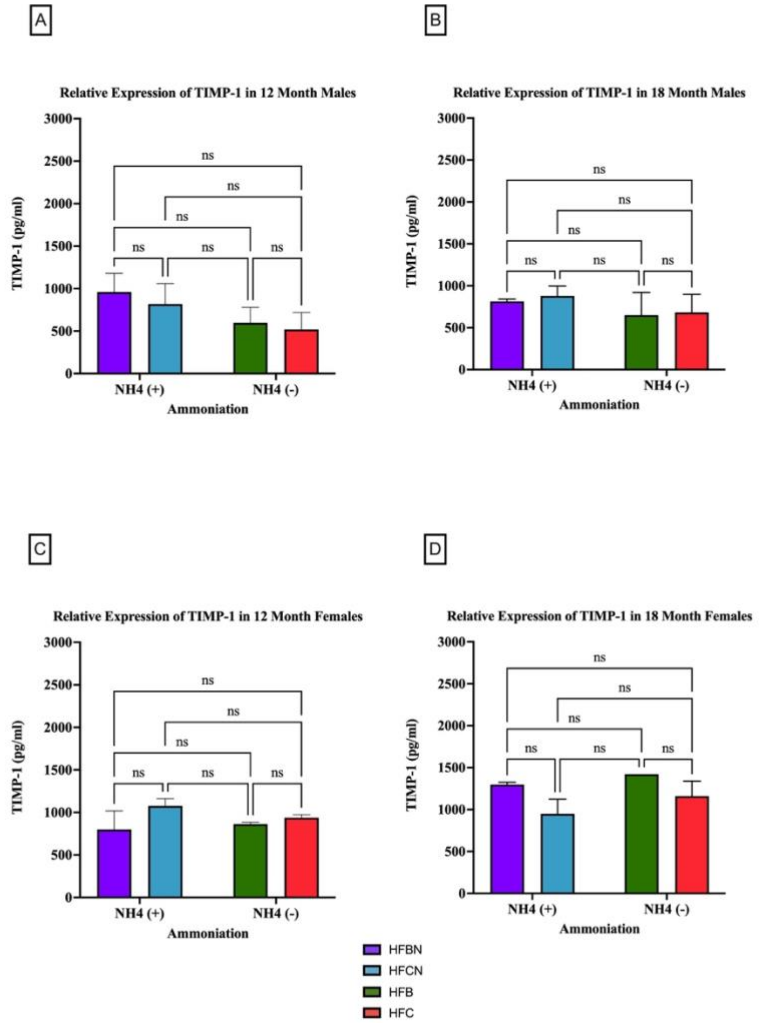

Figure S3. The effect of dietary protein ± AHE on TIMP-1 protein levels for male and female C3H/HeJ mice at 12- and 18-month. **(A)** Male mice at the 12-months. **(B)** Male mice at the 18-months. **(C)** Female mice at the 12-months. **(D)** Female mice at the 18-months. The colors represent diet groups as follows: purple = HFBN, blue = HFCN, green = HFB, and red = HFC. No **s**tatistical significance was observed.

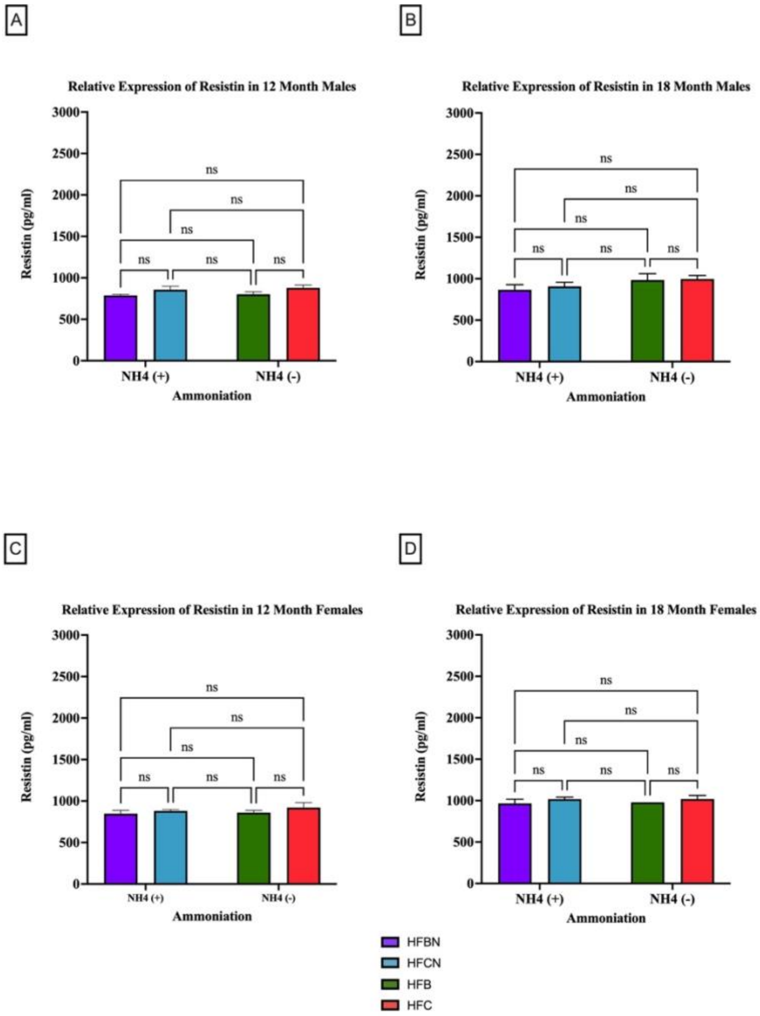

Figure S4. The effect of dietary protein ± AHE on resistin protein levels for male and female C3H/HeJ mice at 12- and 18-month. **(A)** Male mice at the 12-months. **(B)** Male mice at the 18-months. **(C)** Female mice at the 12-months. **(D)** Female mice at the 18-months. The colors represent diet groups as follows: purple = HFBN, blue = HFCN, green = HFB, and red = HFC. No **s**tatistical significance was observed.

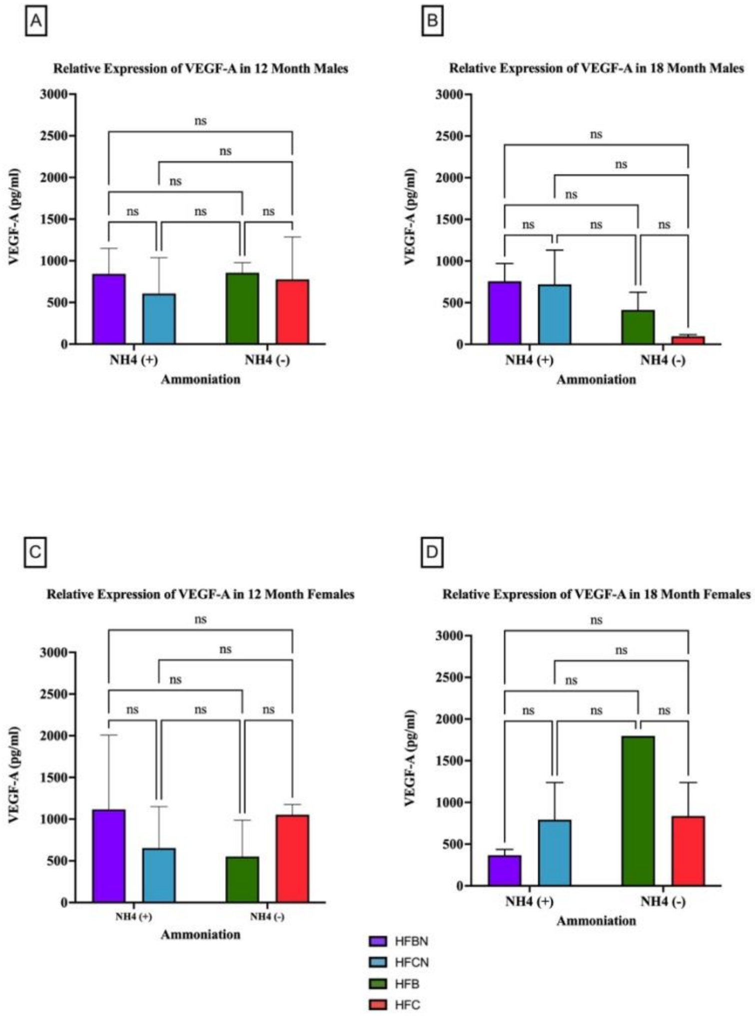

Figure S5. The effect of dietary protein ± AHE on VEGF-A protein levels for male and female C3H/HeJ mice at 12- and 18-month. **(A)** Male mice at the 12-months. **(B)** Male mice at the 18-months. **(C)** Female mice at the 12-months. **(D)** Female mice at the 18-months. The colors represent diet groups as follows: purple = HFBN, blue = HFCN, green = HFB, and red = HFC. No **s**tatistical significance was observed.

Table S6. Total Dietary Components and Formulations

	D20013102	D20013101	D20013103	D20013104	D20013105	D20013106
Product #	*11 kcal% Fat*<br>*Control Diet*	*11 kcal% Fat*<br>*Control Diet*	*46 kcal% Fat*<br>*High-Fat Diet*	*46 kcal% Fat*<br>*High-Fat Diet*	*46 kcal% Fat*<br>*High-Fat Diet*	*46 kcal% Fat*<br>*High-Fat Diet*
	Non-AHE Casein<br>*(CC)*	*AHE-Casein*<br>*(CCN)*	*AHE Beef (HFBN)*	*non-AHE Beef (HFB)*	*AHE Casein (HFCN)*	*non-AHE Casein (HFC)*
Ingredient (g)						
Casein	200	0	0	0	0	200
Casein, "pH-Enhanced"	0	200	0	0	200	0
Beef, Cooked, Freeze Dried, AHE	0	0	352.42	0	0	0
Beef, Cooked, Freeze Dried, non-AHE	0	0	0	293.64	0	0
L-Cystine	3	3	3	3	3	3
Corn Starch	452.2	452.2	68.68	68.46	72.8	72.8
Maltodextrin 10	75	75	100	100	100	100
Sucrose	175.21	175.21	175.21	175.21	175.21	175.21
Cellulose	50	50	45.07	48.53	50	50
Soybean Oil	25	25	25	25	25	25
Lard	0	0	0	0	0	0
Beef Fat, Bunge	20	20	23.28	78.76	177.5	177.5

Mineral Mix S10026 (No Ca, P, K)	0	0	0	0	0	0
Mineral Mix S10026A (No Ca, P, K, Na, Cl)	5	5	5	5	5	5
Dicalcium Phosphate	13	13	13	13	13	13
Calcium Carbonate	5.5	5.5	5.5	5.5	5.5	5.5
Potassium Citrate, 1 H2O	16.5	16.5	16.5	16.5	16.5	16.5
Sodium Chloride	2.546	2.546	1.088	0.87	2.546	2.546
Vitamin Mix V10001	10	10	10	10	10	10
Choline Bitartrate	2	2	2	2	2	2
Cholesterol	0.6	0.6	0	0.02	0.49	0.49
Yellow Dye #5, FD&C	0	0	0	0.025	0.05	0
Red Dye #40, FD&C	0.05	0.05	0.025	0.025	0	0
Blue Dye #1, FD&C	0	0	0.025	0	0	0.05
Total	1055.606	1055.606	845.798	845.54	858.596	858.596
gm						

Protein	179.0	179.0	179.0	179.0	179.0	179.0
Carbohydrate	712.4	712.4	358.0	358.0	358.0	358.0
Fat	47.4	47.4	204.9	204.9	204.9	204.9
Fiber	50.0	50.0	50.0	50.0	50.0	50.0
Cholesterol	0.66	0.66	0.66	0.66	0.66	0.66
gm%						
Protein	17.0	17.0	21.2	21.2	20.8	20.8
Carbohydrate	67.5	67.5	42.3	42.3	41.7	41.7
Fat	4.5	4.5	24.2	24.2	23.9	23.9
Fiber	4.7	4.7	5.9	5.9	5.8	5.8
Cholesterol	0.062	0.062	0.08	0.08	0.08	0.08
kcals						
Protein	716.0	716.0	716.0	716.0	716.0	716.0
Carbohydrate	2849.6	2849.6	1432.1	1432.1	1432	1432
Fat	426.6	426.6	1844	1844.0	1844.1	1844.1
Total	3992.2	3992.2	3992.1	3992.1	3992.1	3992.1
kcal%						
Protein	18	18	18	18	18	18
Carbohydrate	71	71	36	36	36	36
Fat	11	11	46	46	46	46
Total	100	100	100	100	100	100
kcal/gm	3.8	3.8	4.7	4.7	4.6	4.6
Linoleic Acid, gm/kg Diet *(NRC Requirement is 6.8)*	12.9	12.9	20.8	21.3	21.9	21.9
Arginine, gm/kg Diet *(NRC*	5.6	5.6	13	13.6	6.9	6.9

Requirement is 3.0)						
Histidine, gm/kg Diet *(NRC Requirement is 2.0)*	4.3	4.3	6.2	6.8	5.2	5.2
Isoleucine, gm/kg Diet *(NRC Requirement is 4.0)*	7.1	7.1	8.7	9.2	8.7	8.7
Leucine, gm/kg Diet *(NRC Requirement is 7.0)*	14.9	14.9	15	15.8	18.3	18.3
Valine, gm/kg Diet *(NRC Requirement is 5.0)*	8.7	8.7	9.4	9.8	10.7	10.7
Threonine, gm/kg Diet *(NRC Requirement is 4.0)*	6.7	6.7	8.2	8.6	8.3	8.3
Lysine, gm/kg Diet *(NRC Requirement is 4.0)*	12.3	12.3	16.2	17.2	15.1	15.1
Methionine, gm/kg Diet *(NRC Requirement is 5.0; half can be Cystine)*	4.7	4.7	4.8	5.5	5.8	5.8
Cystine, gm/kg Diet *(NRC Requirement is - see above)*	3.0	3.0	5.5	5.9	3.6	3.6

Phenylalanine, gm/kg Diet *(NRC Requirement is 7.6; half can be Tyrosine)*	7.9	7.9	7.6	8	9.7	9.7
Tyrosine, gm/kg Diet *(NRC Requirement is - see above)*	8.5	8.5	6.2	6.5	10.5	10.5
Tryptophan, gm/kg Diet *(NRC Requirement is 1.0)*	2.0	2.0	2.2	2.4	2.4	2.4
Sodium (mg/kg diet)	979	979	1221	1222	1203	1203
Sodium (mg/3982.5 kcal)	1033	1033	1033	1033	1033	1033
